# Seasonal dynamics of free-living (FL) and particle-attached (PA) bacterial communities in a plateau reservoir

**DOI:** 10.3389/fmicb.2024.1428701

**Published:** 2024-07-19

**Authors:** Yang Yang, Chen Chen, Kai Yao, Hans-Peter Grossart

**Affiliations:** ^1^School of Life Sciences, Guizhou Normal University, Guiyang, China; ^2^Leibniz-Institute of Freshwater Ecology and Inland Fisheries, Berlin, Germany; ^3^Potsdam University, Potsdam, Germany

**Keywords:** Yungui plateau, temporal bacterial dynamics, co-occurrence network, Wujiangdu reservoir, deterministic or stochastic processes

## Abstract

In terms of lifestyle, bacterioplankton can be classified as free-living (FL) and particle-attached (PA) forms, and both play essential roles in biogeochemical cycling in aquatic ecosystems. Structure, distribution, and community assembly of FL and PA bacteria in plateau riverine waterbodies are largely unknown. Therefore, we explored the seasonal dynamics of FLand PA bacterial communities in the Wujiangdu reservoir, Yungui Plateau using 16S rRNA gene high-throughput sequencing. Results revealed there was a significant environmental heterogeneity in Wujiangdu reservoir seasonally. The dominant phylum was *Actinomycetota* for FL and *Pseudomonadota* for PA bacteria. Species richness and diversity was higher in autumn and winter compared to spring and summer. In general, PA diversity was greater than FL, but with some temporal variations. Species turnover was the major contributor to β-diversity of both FL and PA lifestyles, and significant differences were noticed between FL and PA bacterial community composition. Distinct co-occurrence network patterns implied that more connections exist between FL bacteria, while more complex PA networks were in parallel to their greater diversity and stronger interactions in biofilms on particles. Dispersal limitation was the major driving force for both FL and PA bacterial community assembly. Deterministic processes were of relatively low importance, with homogeneous selection for FL and heterogeneous selection for PA bacteria. Temperature was the most important environmental driver of seasonal bacterial dynamics, followed by nitrate for FL and Secchi depth for PA bacteria. This study allows for a better understanding of the temporal variability of different bacteria lifestyles in reservoirs in the vulnerable and rapidly changing plateau environment, facilitating further microbial research related to global warming and eutrophication.

## 1 Introduction

Based on lifestyle, aquatic bacteria can be classified as free-living (FL) and particle-attached (PA; Grossart, 2010). In aquatic systems, FL and PA bacteria are considered as interacting assemblages, and switching of lifestyles is a key factor for bacterial particle remineralization (Villalba et al., 2022). Particles are enriched in carbon and nutrients compared to their surrounding water, hosting PA communities which differ from their FL counterparts. FL bacteria, generally with small genomes and high expression of membrane transporter genes, can better adapt to low nutrient conditions than PA bacteria (Smith et al., 2002; Satinsky et al., 2014). On the contrary, PA bacteria prefer anoxic and nutrient-abundant habitats (Grossart et al., 2007; Rosel and Grossart, 2012; Villalba et al., 2022). PA bacteria are generally more active and more diverse in metabolic functions (Crespo et al., 2013; Ortega-Retuerta et al., 2013).

Seasonal changes in bacterioplankton have been reported as community shifts of closely related strains switching among seasons (Ward et al., 2017). Thereafter, both regional and local factors contribute to the distribution and turnover of microbial communities (Logue et al., 2011). Temporal change was observed for both FL and PA bacteria among seasons. In case of a river-ocean transition zone, spatial variability overwhelmed the observed temporal changes (Fortunato et al., 2012). Critical environmental factors for such seasonal patterns of bacterial dynamics were physicochemical factors, associated with both latitude and distance. Different bacterial populations have distinctive traits in metabolism and nutrient preferences. Besides, biological interactions (e.g., between bacteria and between bacteria and phytoplankton, fungi and protist grazers) can substantially influence the temporal dynamics. Bacterioplankton showed several adaptations in relation to their micro-habitat heterogeneity (Grossart, 2010; Enke et al., 2018; Villalba et al., 2022). For lakes, it has been found that bacterial community composition is highly system-specific (Konopka et al., 1999) and vary over time (Yannarell et al., 2003; Zwisler et al., 2003). Similar to several other studies in Poyang Lake, a clear ecological differentiation between FL and PA bacteria were found for this flood-plane lake (Ma et al., 2022). Temperature (Yannarell and Triplett, 2004), water chemistry (e.g., pH, DO, alkalinity, DOC and nutrients etc.; Crump et al., 2003; Allgaier and Grossart, 2006) and phytoplankton composition (Simek et al., 2002) have been noted to be associated with such temporal variability of bacterial lifestyle. However, to our knowledge, seasonality of different bacterial lifestyles in riverine plateau reservoirs and their environmental drivers remain largely unknown.

Not only composition and diversity of FL and PA bacteria can differ seasonally, but also in their assembly processes. Thereby, deterministic and stochastic processes constitute two complementary mechanisms for microbial community assembly (Zhou and Ning, 2015; Langenheder and Lindstrom, 2019). Whereas deterministic processes correspond to species sorting driven by biotic and abiotic factors, including homogeneous selection (HoS) and heterogeneous selection (HeS), stochastic processes comprise random events such as ecological drift, homogenizing dispersal (HoD) and dispersal limitation (DL; Martiny et al., 2011). Moreover, random fluctuations in birth and death rates as well as species gaining or loss by chance can contribute to community turnover (Vellend, 2016).

Rivers connect terrestrial and marine environments, e.g., via transport of organisms and nutrients. Due to the small bacterial cell size and high hydrodynamic force in rivers, homogenizing dispersion might result in similar communities among locations. Thereby, versality and plasticity in bacterial metabolism and functions enhance the ability of bacteria to occur in different environment, with a potential to override environmental selection pressure. Furthermore, the unidirectional riverine water flow might cause a ‘mass effect’, changing bacterioplankton distribution. Despite numerous studies on aquatic bacterial communities, temporal and spatial dynamics of microbial communities in rivers are in general poorly understood. Riverine reservoirs in the plateau region, have low water retention time and little is known about the dynamic distinction between FL and PA bacteria lifestyles in such a riverine reservoir system. The Wujiangdu reservoir is part of the Wujiang river, which is a southern tributary of the Yangtze River. It is 1,037 km long and a major power source for China’s massive west-to-east power transmission project. In our study, we assumed that (1) FL and PA bacterial lifestyle lead to differences in community composition, diversity and assembly processes; (2) patterns of FL and PA lifestyle dynamics vary seasonally; (3) environmental factors modulating the temporal changes of both FL and PA bacterial lifestyles differ in this riverine reservoir with heavy flow. This study aims to provide insights into both seasonal dynamics and community assembly processes of FL vs. PA bacteria in the plateau Wujiangdu reservoir.

## 2 Materials and methods

### 2.1 Sampling locations and field work

**Figure 1:**
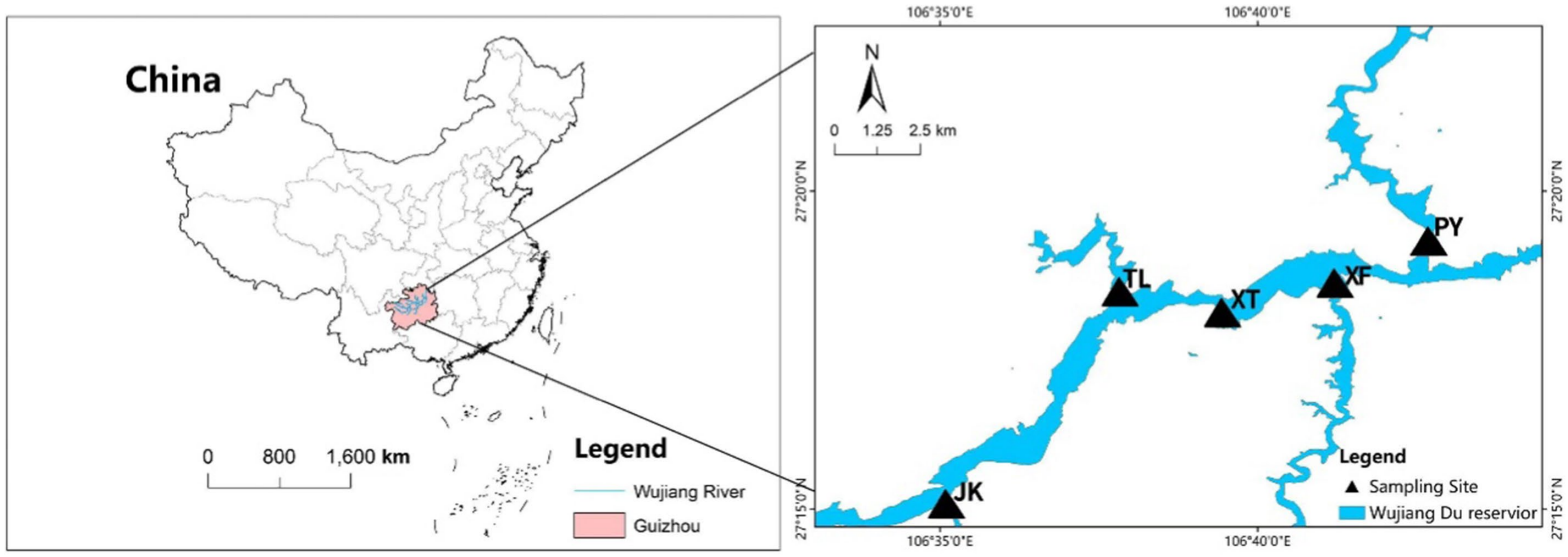
Sampling locations in the Wujiangdu reservoir, China (5 sites: JK, TL, XT, XF and PY).

The Wujiangdu reservoir is located in the lower basin of the Wujiang River, Guizhou Province, a typical karst area. It was built in 1979 with a watershed of 2.78 × 10^3^ km^2^, a volume of 23 × 10^8^ m^3^, and an average depth of 154 m. The average water residence time is relatively low for the size of the reservoir, i.e., only 53 days. Five sampling locations were selected, i.e., at the intersection of the tributaries along the Wujiang mainstream (JK, TL, XF, and PY), whereby the XT site comprise a relative sheltered place (Figure 1).

Samples were collected from September 2021 to August 2022. Seasons were classified as spring (March to May), summer (June to August), Autumn (September–November), and Winter (December–February). Water temperature (Temp), dissolved oxygen (DO), pH and conductivity (Cond) were quantified *in situ* using a YSI probe (HANNA HI98194). Secchi depth (SD) was measured by using a Secchi disk. One liter of water was used for chemical water analysis, including total nitrogen (TN), total phosphorus (TP), total dissolved nitrogen (TDN), total dissolved phosphorus (TDP), nitrate (NO_3_^−^-N), nitrite (NO_2_^−^-N), ammonium (NH_4_^+^-N), phosphate (PO_4_^3−^-P), dissolved silicate (DSi), Total Suspended Solid (TSS), and chlorophyll a (Chl *a*) according to standard method (APHA [American Public Health Association], 2012). A second one-liter subsample was stored in an icebox for DNA extraction in the lab. Samples were transported to the lab within 4 h. FL and PA bacterial communities were separated by sequential filtration, i.e., 0.6 L water was filtered through a 5.0 μm pore-size filter (Isopore Millipore) constituting the PA fraction, and the filtrate was then filtered through a 0.22 μm pore-size filter constituting the FL fraction. All filters were preserved at −80°C until further DNA analysis.

### 2.2 DNA extraction and sequencing

DNA extraction was conducted using the MagaBio Soil and Feces Genomic DNA Purification Kit (Bori Technology, China) following the manufacturer’s instructions. The V4-V5 region of 16S rRNA gene was amplified using the primer 515F (5’-GTGCCAGCMGCCGCGGTAA-3′) and 907R (5’-CCGTCAATTCMTTRAGTTT-3′). PCR products were pooled and purified using the QIAquick PCR purification kit (QIAGEN) and quantified via a NanoDrop ND-2000 (Thermo Scientific). Then high-throughput sequencing PE250 was performed on the Illumina Nova 6,000 platform (Magigene Biotechnology Co., China). Quality filtering on the pair-end raw reads was performed according to the Trimmomatic quality-control process. Paired end, clean reads were merged using FLASH. The DADA2 pipeline was applied for paired-end sequences in the fastq files to provide the amplicon sequence variant (ASV) table for each sample. Taxonomic information was assigned to ASVs, used for subsequent statistical analysis.

### 2.3 Statistical analyses

Diversity indices were calculated for FL and PA bacteria, including richness, Simpson, Shannon, Pielou’s evenness, abundance coverage estimator (ACE) and phylogenetic diversity (PD) using the ‘vegan’ package in R. β-diversity, based on Bray–Curtis dissimilarity was used to detect the variations in community structures. Analysis of variance (ANOVA) was performed to test for significant difference between different bacterial lifestyles and seasons. Analysis of similarity (ANOSIM) and permutational multivariate analysis of variance (PERMANOVA) were applied to test for compositional differences between seasons. PERMDISP was used to test the dispersion extent of communities within seasons (Anderson et al., 2008). Non-parametric multivariate dimensional scaling (NMDS) based on Bray–Curtis dissimilarity was used to detect the distribution of FL and PA bacterial communities. Redundancy analysis (RDA) was performed to analyze the relationship between environmental factors with FL and PA bacterial community, respectively. Mantel tests were performed to test the correlation between bacterial community dissimilarity with geographic distance and environmental dissimilarity using Spearman’s correlation ecoefficiency.

Bacterial community assembly processes were evaluated via the null model (Stegen et al., 2013) to quantify the relative contribution of deterministic and stochastic processes (Kajan et al., 2023). Bray-Curtis-based Raup-Crick (RC_bray_) was calculated using the ‘picante’ package. βNTI values < −2, refer to a significantly lower than expected phylogenetic turnover (homogeneous selection, HoS), while βNTI values >2 indicate a significantly higher than expected phylogenetic turnover, variable or heterogeneous selection (HeS). Values of |βNTI| < 2 and RC_bray_ > 0.95 reflect the relative importance of dispersal limitation (DL), whereas |βNTI| < 2 and RC_bray_ < − 0.95 refer to the primary contribution of homogenizing dispersal (HoD). In contrast, values of |βNTI| < 2 and | RC_bray_ | < 0.95 represent the effect of undominated processes, implying no single process drives community assembly (Ning et al., 2020).

An ecological co-occurrence network was constructed to evaluate interaction patterns of FL and PA bacterial communities. ASVs with a > 1/3 occurrence frequency were selected to construct the network. All pairwise Spearman’s rank correlations (r) were calculated, and significant correlations (*p* < 0.05) with a |r| > 0.6 were considered to be effective and subjected to subsequent analyses using the ‘igraph’ package (Csárdi et al., 2024). A network graph was plotted using Gephi 1.0 (Bastian et al., 2009).

Co-occurrence patterns of FL and PA lifestyles were analyzed to reveal the interactions among bacterial taxa based on network theory using the molecular ecological network analysis (MENA) pipeline. Only ASVs with an occurrence >50% were selected for the analysis. Spearman’s correlation coefficients |r| > 0.80 with *p* < 0.01were chosen for co-occurrence network construction. The topological roles of each OTUs can be defined according to Zi (the connectivity of node I within modules) and Pi (the connectivity of node I among modules) values (Guimerà and Amaral, 2005). Nodes could be classified into 4 categories: (1) network hubs (Zi > 2.5, Pi >0.62); (2) module hubs (Zi > 2.5, Pi ≤0.62); (3) connectors (Zi ≤ 2.5, Pi >0.62); and (4) peripherals (Zi ≤ 2.5, Pi ≤0.62; Zhou et al., 2011).

## 3 Results

### 3.1 Seasonal variation of environmental variables

**Figure 2:**
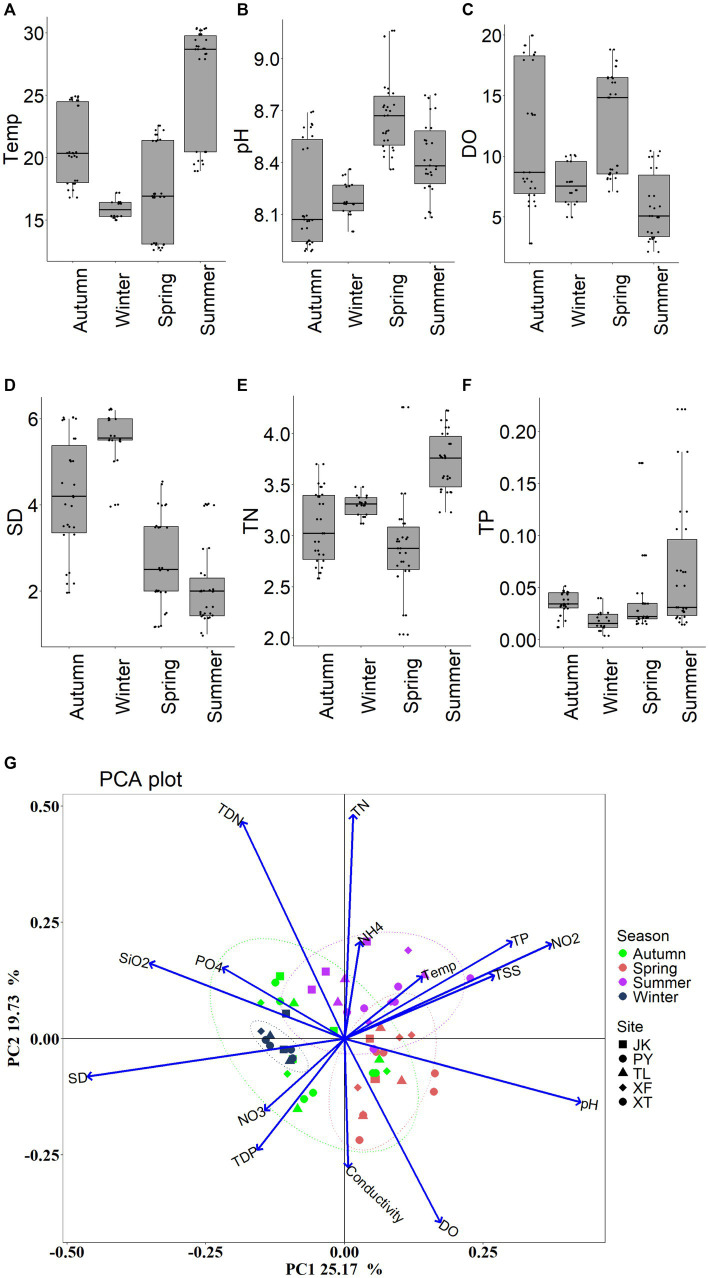
Seasonal variations of environmental variables. **(A)** Temp: water temperature; **(B)** TSS: total suspended solid; **(C)** SD: Secchi depth; **(D)** pH; **(E)** TN: total nitrogen; **(F)** TP: total phosphorus; and **(G)** PCA on all measured environmental factors.

There were clear temporal variations of environmental factors (Figure 2). ANOVA tests revealed that there were significant temporal differences in Water Temperature (Temp), TSS, SD, pH, TN and TP (*p* < 0.01). In the PCA biplot, Temp, TP, NO_2_^−^-N, TSS, pH was positively correlated with PC1 (with a total variation explained of 25.17%), PO_4_^3−^-P, DSi, and SD were negatively correlated with PC1. TN, TDN, NH_4_^+^-N, NO_3_^−^-N, TDP, DO and Conductivity (Cond) were associated with PC2 (accounting for 19.73% of the total variations).

### 3.2 FL and PA bacterioplankton community composition

**Figure 3:**
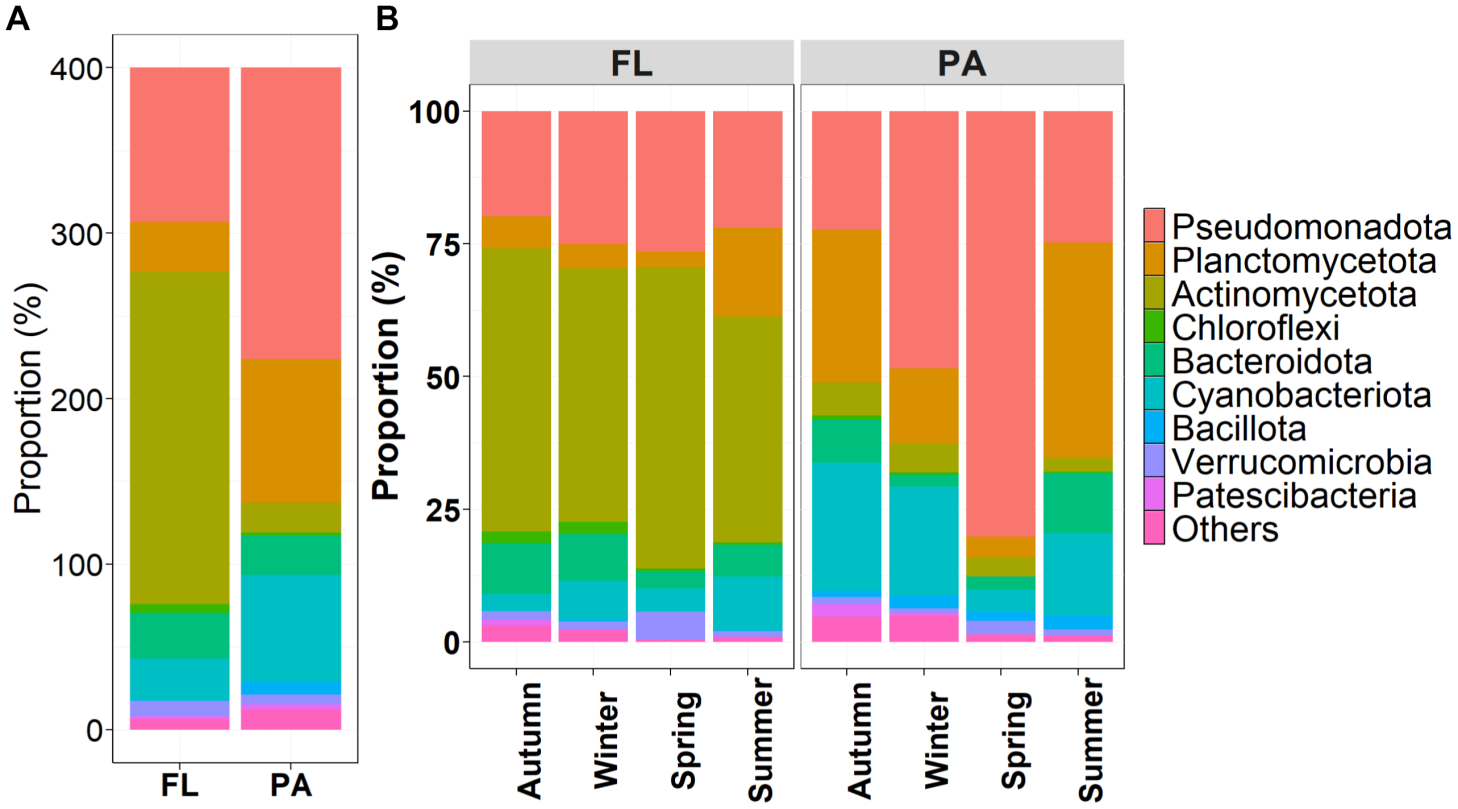
Variations in relative abundance of **(A)** the top 10 phyla of FL and PA bacterial communities, and **(B)** across seasons in the Wujiangdu reservoir.

At the phylum level, FL and PA bacteria community composition (BCC) exhibited different dynamics across seasons (Figure 3A). Dominant phyla of the FL lifestyle were *Actinomycetota* (50.13%) and *Pseudomonadota* (23.35%), while for the PA lifestyle, *Pseudomonadota* (43.98%), *Planctomycetota* (21.78%), and *Cyanobacteriota* (16.07%) constituted the dominant phyla. Thereby, the relative abundance of dominant phyla within each lifestyle community varied seasonally (Figure 3B). BCC_PA_ exhibited higher variations than BCC_FL_. From autumn to the next spring, *Pseudomonadota* increased, *Planctomycetota* and *Cyanobacteriota* decreased in both BCC_FL_ and BCC_PA_. Compared to other seasons, in summer BCC_FL_ revealed less *Actinomycetota* but more *Planctomycetota*. In summer BC_PA_, *Planctomycetota* (40.47%) became the predominant phylum with a subdominance of *Pseudomonadota* (24.80%) in Figure 3.

### 3.3 FL and PA bacterial community (BC_FL_ and BC_PA_) diversity dynamics

**Figure 4:**
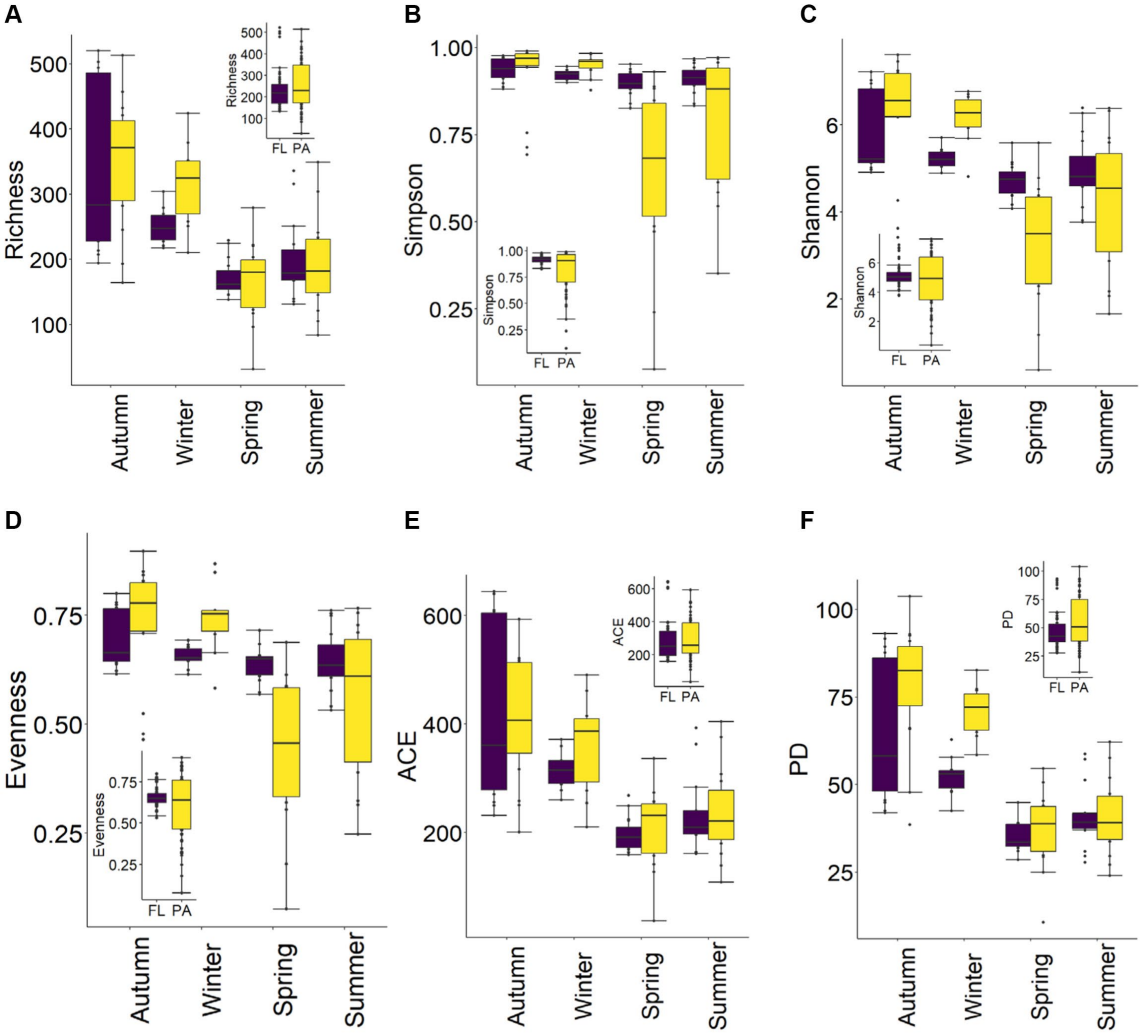
Seasonal variations of α-diversity of BC_FL_ and BC_PA_ in the Wujiangdu Reservoir **(A)** ASV richness; **(B)** Simpson; **(C)** Shannon; **(D)** Pielou’s Evenness; **(E)** ACE; **(F)** PD.

Overall, BC_PA_ diversity [ASV richness, Simpson, and Shannon diversity indices, evenness, ACE and Phylogenetic diversity (PD)] was higher than for BC_FL_. Yet, there were different patterns in BC_FL_ vs. BC_PA_ diversity among seasons. Temporal variations in diversity revealed that ASV richness increased from spring to summer and was highest in autumn. Generally, BC_FL_ and BC_PA_ diversity in autumn and winter were higher than in spring and summer. In autumn and winter, BC_PA_ diversity was greater than for BC_FL_, whereas in spring and summer, BC_FL_ diversity was greater than for BC_PA_ (Figure 4). ANOVA on diversity indices revealed that significant differences occurred among seasons for BC_FL_ and BC_PA_ (*p* < 0.05). In spring, Simpson and Shannon diversity indices and evenness showed significant differences between BC_FL_ and BC_PA_ (*p* < 0.01). While there were no significant differences in diversity indices in summer and autumn, except a weak significant difference for Simpson in summer and PD in autumn (0.01 < *p* < 0.05). In winter, ASV richness, Simpson, and Shannon diversity indices and evenness of BC_FL_ and BC_PA_ exhibited significant differences (*p* < 0.05).

**Figure 5:**
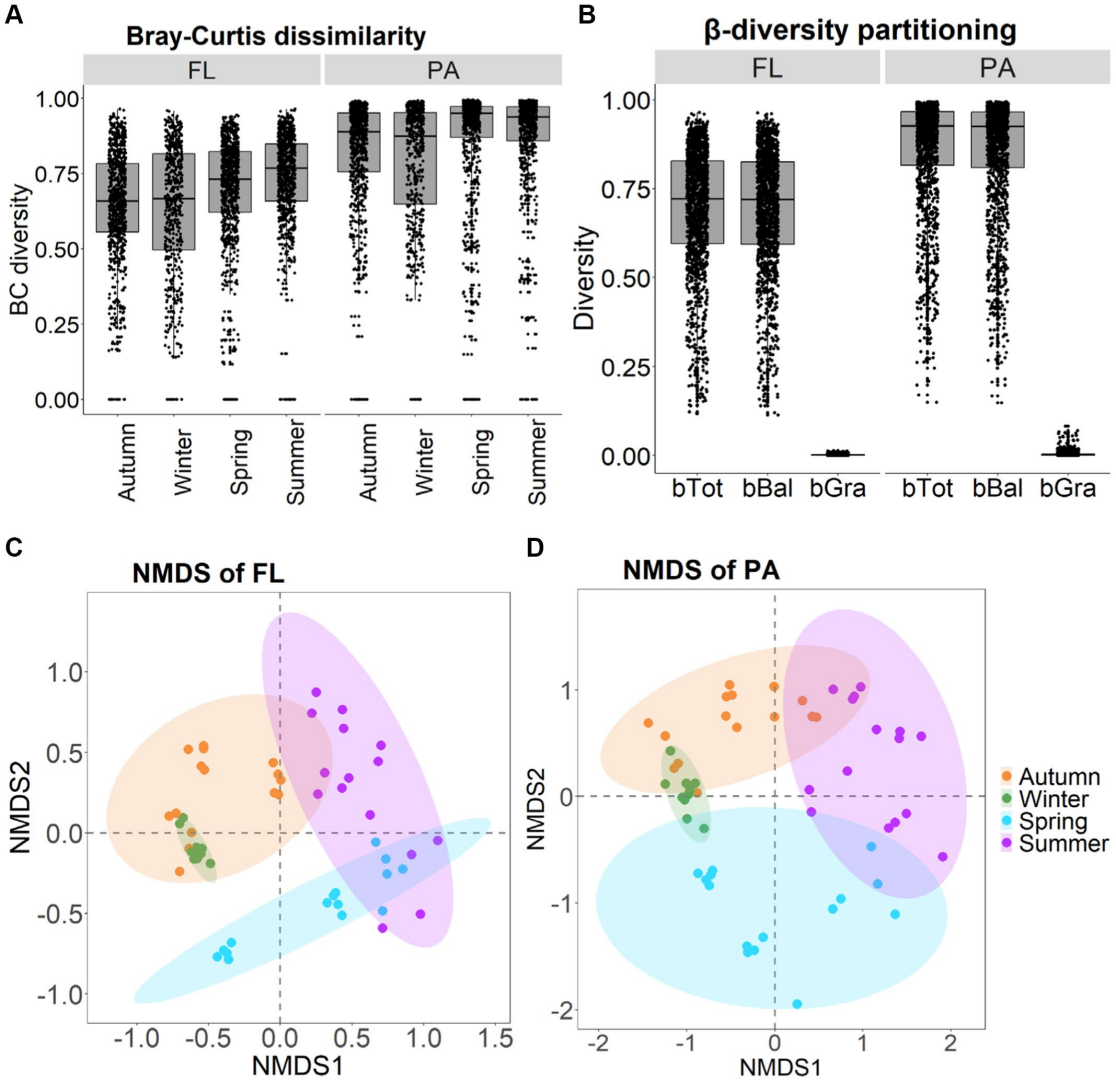
Seasonal variation in Bray-Curtis dissimilarity of BCFL and BCPA **(A)**; β-diversity partitioning for BCFL and BCPA **(B)**; and NMDS plot of BCFL **(C)** and BCPA **(D)**.

β-diversity of BC_FL_ and BC_PA_ showed similar patterns with the order of summer > autumn > spring > winter as indicated by Bray–Curtis dissimilarity (Figures 5A,B). β-diversity exhibited seasonal variations, which were highest in summer and lowest in winter. For BC_FL_, β-diversity in spring was higher than in autumn. Whereas for BC_PA_, β-diversity in spring was lower than in autumn. Significant differences between BC_FL_ and BC_PA_ were found within the four seasons (*p* < 0.001). In addition, for BC_FL_ and BC_PA_ separately, significant differences were detected between seasons (*p* < 0.001) (Figures 5C,D). Moreover, a significant dispersion of bacterial communities occurred in autumn and winter as revealed by PERMDISP tests (*p* < 0.01; Table 1). A pairwise comparison implies that for BC_FL_ significant differences occurred between winter and other seasons (*p* < 0.01), and between summer and autumn (*p* = 0.02). For BC_PA_ lifestyle, significant differences occurred between winter and other seasons. β-diversity partitioning showed that the primary contributor derived from balanced variation in abundance (bBal) instead of abundance gradients (Figure 5B).

**Table 1:** PerMANOVA, ANOSIM and PERMDISP tests between BC_FL_ and BC_PA_ within each season.

Season	Adonis	ANOSIM	PERMDISP
Spring	0.001	0.001	0.09
Summer	0.001	0.001	0.11
Autumn	0.001	0.001	<0.001
Winter	0.001	0.001	0.003

### 3.4 Network analysis of BC_FL_ and BC_PA_

**Figure 6:**
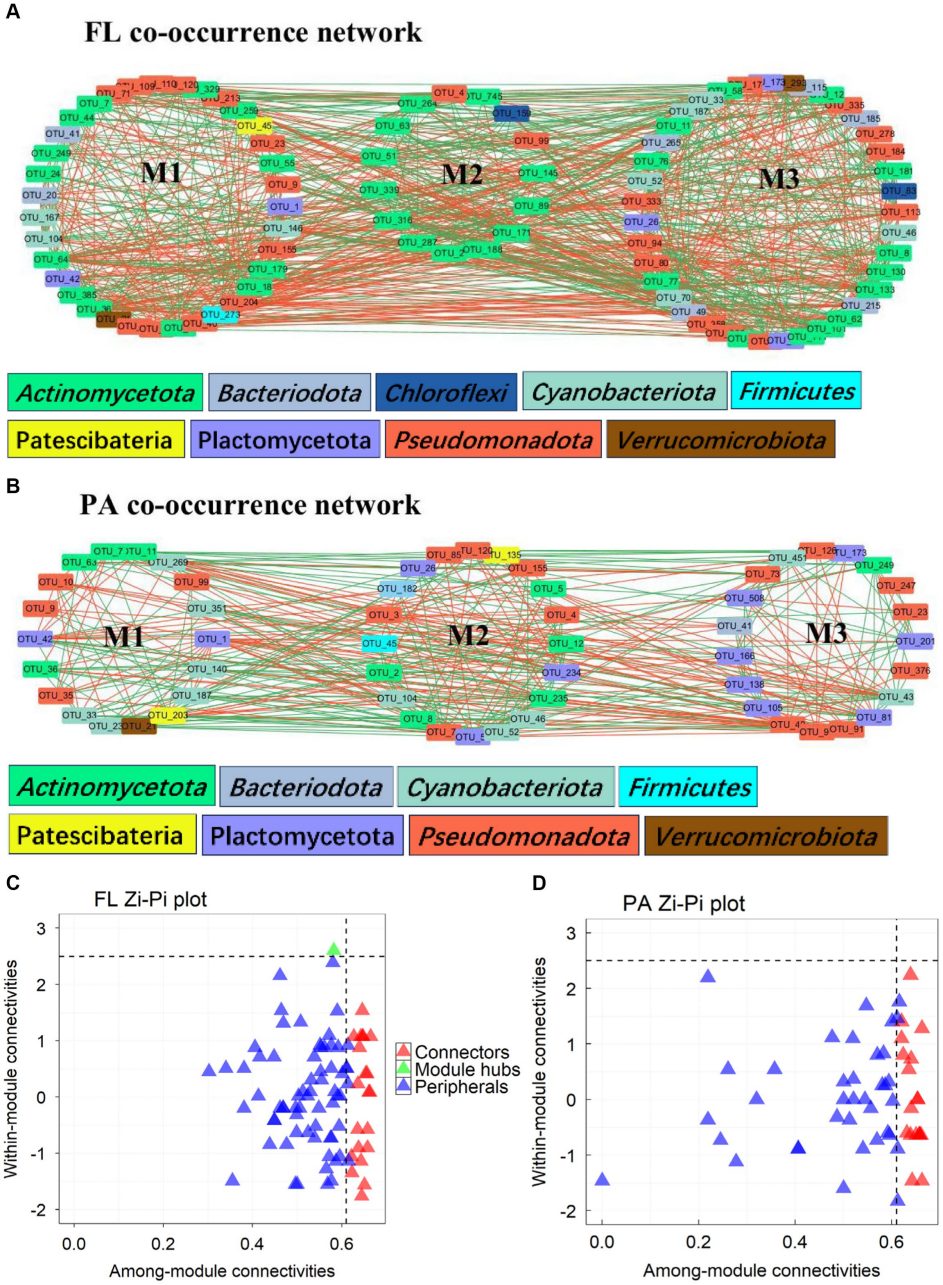
Network analysis of BC_FL_ **(A)** and BC_PA_ **(B)**; Zi-Pi plot of ASVs in BC_FL_ **(C)** and BC_PA_ **(D)** networks (the color of nodes refers to the phylum of the OTU; green edges: positive correlation; red edges: negative correlation; M1-M3 refers to modules in each network; Zi: within-module connectivity; Pi: among-module connectivity).

Networks of BC_FL_ had more nodes, edges and higher values of AD and GD than those of BC_PA_, and also more keystone taxa (Table 2). BC_PA_ networks, however, showed a higher modularity (Figures 6A,B). Keystone taxa in network analyses refer to modules and connectors. In BC_FL_ networks, one module hubs (ASV_70) and 22 connectors (Figure 6C). Module hub ASV_70 was *Synechococcus* from *Cyanobacteria*. In BC_PA_ networks, 20 connectors were observed (Figure 6D). ASV_11 (*Actinomycetota*), ASV_21 (*Verrucomicrobiota, Luteolibacter*), and ASV_36 (*Actinomycetota, Ilumatobacteraceae*) were connectors for both BC_FL_ and BC_PA_ networks. The taxonomic information of all connectors and module hubs is given in Supplementary Table S1.

**Table 2:** Topological attributes of co-occurrence network of BC_FL_ and BC_PA_ bacteria in the Wujiangdu reservoir.

	Node	Edge	Modularity	Density	AD	avgPL	avgCC
FL	90	1396/432	0.15	0.35	31.02	1.67	0.46
PA	57	437/199	0.20	0.27	15.33	1.78	0.41

AD, average degree; avgPL, average path; avgCC, average clustering coefficient.

### 3.5 Relationship between environmental factors and BC_FL_ and BC_PA_ bacterial communities

**Figure 7:**
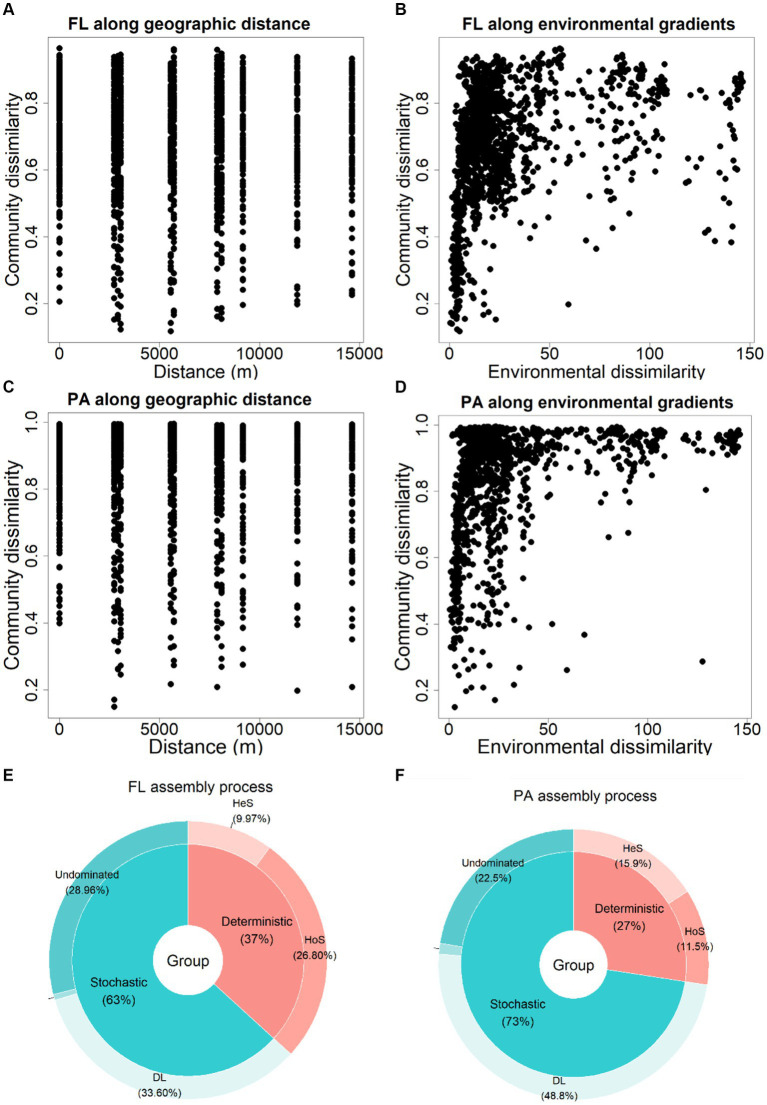
Relationship between community similarity (Bray-Curtis distance from the ASV table) of BC_FL_ and BC_PA_ related to **(A,C)** geographic distance and **(B,D)** environmental similarity; Bacterial community assembly processes for **(E)** BC_FL_ and **(F)** BC_PA_. DL: dispersal limitation, HeS and HoS: heterogeneous and homogeneous selection, respectively.

Neither BC_FL_ nor BC_PA_ community showed significant correlations with geographic distance (*p* = 0.90 and *p* = 0.78 respectively; Figures 7A,C). Yet, significant correlations were noticed between bacterial communities and environmental factors (*p* = 0.001; Figures 7B,D).

Based on community assembly analysis, stochastic processes were the dominant mechanism driving both BC_FL_ (63%) and BC_PA_ (73%; Figures 7E,F). Dispersal limitation (DL) predominated in BC_FL_ (33.6%) community assembly, while DL (48.8%) and undominated processes (28.96%) were the major component in BC_PA_ assembly. There was a relatively higher importance of deterministic processes (mainly homogeneous selection) for BC_FL_ than BC_PA_ (mainly heterogeneous selection).

**Figure 8:**
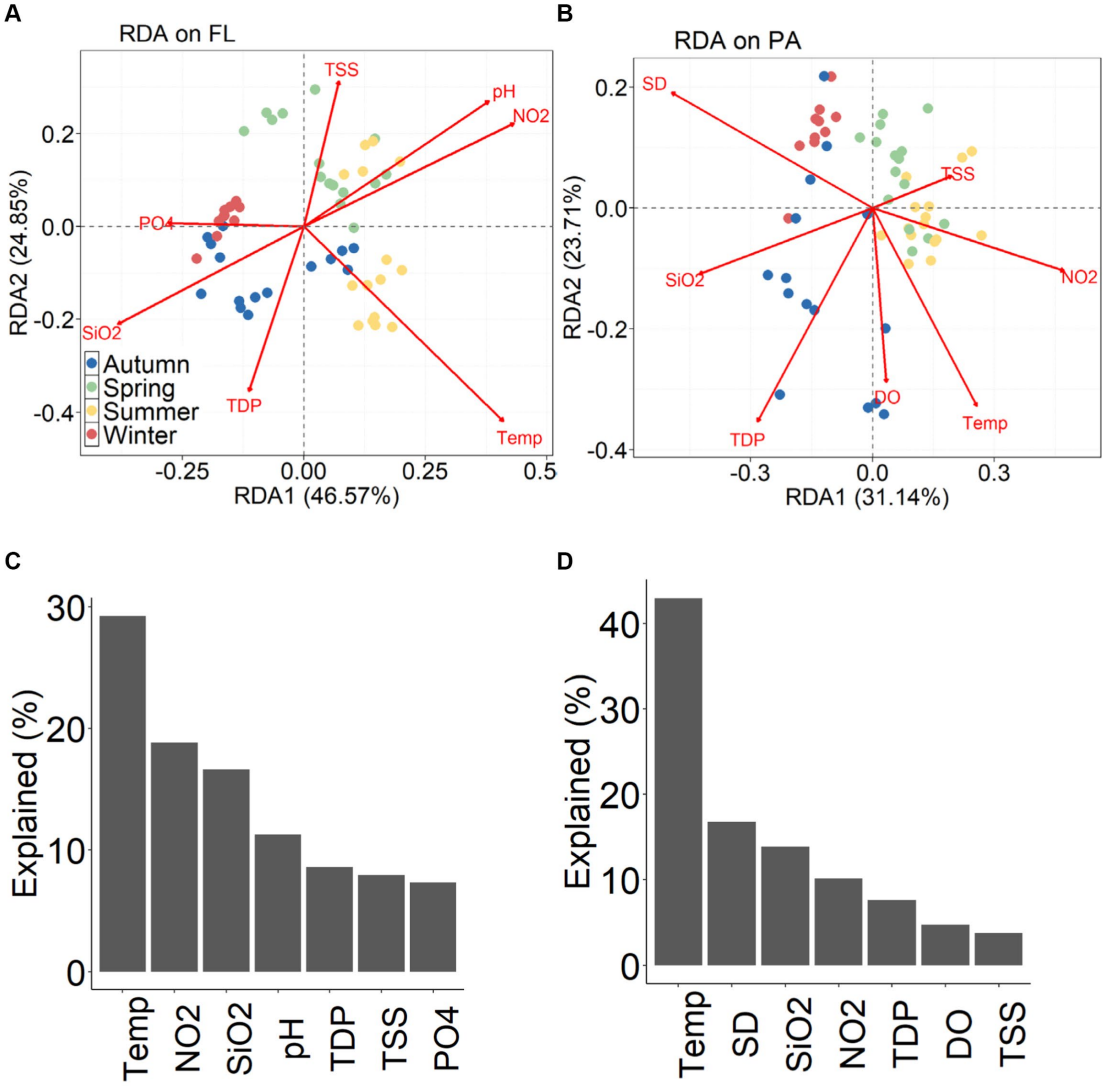
RDA plot **(A)** BC_FL_ and **(B)** BC_PA_ and the percentage of variation explained by individual environmental variables for **(C)** BC_FL_ and **(D)** BC_PA._

The distribution in the RDA biplot indicate the seasonal dynamics of BC_FL_ and BC_PA_ (Figures 8A,B). For BC_FL_ bacteria, the most important environmental factors were water temperature, nitrate, silicate and pH (Figure 8C), while for BC_PA_, water temperature, Secchi depth, and silicate were the primary factors controlling seasonal development (Figure 8D). In each season, correlations between environmental factors and bacterial community structure were revealed by Mantel tests (Supplementary Figure S1). The significant environmental factors varied among different seasons for BC_FL_ and BC_PA_, and between BC_FL_ and BC_PA_.

## 4 Discussion

There was significant temporal environmental heterogeneity, which caused the seasonal variation of both FL and PA bacterial communities. PCA indicates clear temporal dynamic patterns of environmental factors in the Wujiangdu reservoir, whereby the higher TP and TSS in summer, implies a higher particle concentration. Moreover, both parameters exhibited a greater variation in autumn and winter. TSS, which mainly consists of detritus from amorphous aggregation of suspended sediment, can be considered as a proxy for the concentration of particles in the water column (Tang et al., 2010). Moreover, variations in the composition of the particles led to differences in diversity of BC_PA_ in summer (Hollibaugh et al., 2000). TSS play critical roles in controlling the relationships between BC_FL_ and BC_PA_ (Dang and Lovell, 2016). They also promote the desorption rate of PA bacteria and subsequently separate them from the particle (Tang et al., 2017). Generally, high TSS contents are related to high turbulence and high primary production, which also change the particle’s microenvironment (Crump et al., 1999). In winter, the low TSS indicates a low concentration of particles resulting in high SD values.

Composition of BC_FL_ and BC_PA_ differed, with *Actinomycetota* was dominating in BC_FL_ and *Pseudomonadota* in BC_PA_. Individual taxa of *Pseudomonadota* are favored by nutrient addition, which might be attributed to their capacity of degrading more complex organic compounds (Newton and McMahon, 2011). *Actinomycetota* were found to be prevalent in temperate lakes (Allgaier and Grossart, 2006), preferentially in BC_FL_. Moreover, BC_FL_ and BC_PA_ exhibited temporal dynamics, reflected by the relative abundance change of the 10 most dominant phyla. *Pseudomonadota* is the largest phylum of bacteria and they respond quickly to changes in nutrient availability (Nielson et al., 2007; Posch et al., 2007). *Planctomycetota* were abundant in summer and decreased their relative abundance in winter and spring. This is inconsistent with other studies that *Planctomycetota* was correlated with the strong growth of algae and abundant in summer and earlier autumn samples (Burke et al., 2011; Kabore et al., 2020).

The observed variations in BC_FL_ and BC_PA_ diversity, were consistent with previous research in other lakes (Glockner et al., 2000; Belkova et al., 2003; Rosel and Grossart, 2012). We observed the lowest diversity in spring and the highest in autumn for both BC_FL_ and BC_PA_, reflecting the temporal heterogeneity of environmental conditions. The decline in α-diversity in spring might be the result of a homogenizing resource effect. The increased disturbances mix the water and induced the similarity of environment, which decreased the diversification of available niches. Generally, BC_PA_ diversity (including evenness) was greater than BC_FL_ generally. But in spring and summer, BC_FL_ diversity was higher than BC_PA_ indicating high seasonal dynamics. PA bacteria may have a metabolic advantage in the absence of dissolved nutrients in the water column. In addition, the particle could serve as a temporal refuge for bacteria to avoid grazing (Villalba et al., 2022). Thus, at times, it can be an advantage for bacteria to choose the PA lifestyle, which may be indicated by the higher diversity of BC_PA_. Moreover, generalist PA bacteria can adapt flexibly and quickly to environmental changes and thus can easily switch between different lifestyles (Teeling et al., 2012; Villalba et al., 2022). Some BC_PA_ taxa can rapidly attach to particles and utilize organic particles, and their priority might inhibit other taxa of PA bacteria. This competition within BC_PA_ may cause the observed decline in BC_PA_ diversity (Calcagno et al., 2017; Fu et al., 2022). Furthermore, the phylogenetic BC_PA_ diversity seem to be higher than BC_FL_, reflecting the diverse nature of different particles in the water column, especially in autumn. Significant differences in BC_FL_ and BC_PA_ diversity among seasons indicate temporal variations of different bacterial communities not only in terms of changes in abundance, but also community turnover with species gaining and loss.

β-diversity of BC_PA_ was higher than BC_FL_, with most contribution from variations in abundance, indicating community variations attributable to variations in species abundance (species turnover). The diversity-begets-diversity hypothesis (Calcagno et al., 2017) proposes that diversity stimulates further diversification as species interactions get more complex and novel niches become available. This may explain the observed greater variation (β-diversity) for BC_PA_. Generally, spring and summer showed a more determined temporal development. Changes in β-diversity may also reflect differences in environmental heterogeneity between seasons, presumably explaining the observed greater variations in environmental conditions during spring and summer in the Wujiangdu reservoir. Furthermore, there were significant differences in BC_FL_ and BC_PA_ composition among seasons, but between BC_FL_ and BC_PA_ within the same season.

Complexity of the BC_PA_ network was higher than of BC_FL_, as indicated by average Clustering Co-efficiency and path length, confirming the co-occurrence relationships are different between both FL and PA lifestyles. Higher connectivity in BC_PA_ presumably due to more potential ecological niches on particles, lead to higher functional diversity among individual microhabitats (Reshef et al., 2012; Peura et al., 2015). More nodes and edge number with higher average degree (AD) and graphic density (GD) imply more ecological connections in the BC_FL_ network. AD and GD, symbolizing the interaction strength inside a community were greater in BC_PA_ than BC_FL_. Keystone taxa, which have greater impacts on the community structure and functioning, usually exhibit a high closeness centrality, low betweenness centrality score, and high mean degree in microbial networks (Banerjee et al., 2019). Cyanobacteria, represent a keystone taxon in the BC_FL_ network, produce organic substance and cyanotoxins, which constitute a favorable microhabitat for bacteria and thus might select for the growth of specific species (Krzton et al., 2019). This emphasizes their indispensable function in the ecosystem. Further, degradation of organic compounds released by cyanobacteria (Paver et al., 2013) may stimulate the cooperative behavior of bacteria and the interaction between cyanobacteria and bacteria. It has been noticed that cyanobacterial blooms are associated with bacterioplankton communities (Schere et al., 2017; Akins et al., 2018), and has impact on the stability and interactions with bacterial communities.

No significant distance decay patterns were observed in this study. This is most likely due to the relatively small spatial scale and the low number of locations (only 5) we have sampled in this riverine reservoir. Observed seasonal dynamics in BC_FL_ and BC_PA_ implies strong effects of environmental factors on bacterial communities, especially for the PA lifestyle. Bacterial community succession was associated with lake stratification, with increasing divergence during summer in different layers of the stratified water column, and the high convergence when the lake was mixed (Schmidt et al., 2008; Diao et al., 2017). Previously, it has been observed that the diversity of BC_PA_ is significantly higher in the pre than the post-algal bloom (Ren et al., 2023; Van Le et al., 2024). PA bacteria have more transporters and are involved in the decomposition of phytoplankton cell biomass. In contrast, the algal-derived DOM might promote the growth of specific FL bacteria adapted to more oligotrophic environments (Alonso-Saez et al., 2008). From summer to autumn, decreasing temperatures and light affect species turnover and bacterial populations, which represent successful scavengers of semi-labile DOM after summer (Bunse and Pinhassi, 2017). Increasing irradiance and temperature in spring trigger massive growth of both algae and bacteria.

Both BC_FL_ and BC_PA_ bacteria community assembly were primarily driven by stochastic processes, with a relatively higher contribution of deterministic process for BC_FL_. Dispersal limitation (DL) was the major driver for bacterial community assembly, which was consistent with another study of riverine microbial communities (Wang et al., 2019). Dispersal limitation showed a greater relative importance in structuring BC_PA_, which might be because of a higher habitat homogeneity and hydrological connectivity in BC_FL_. The hydrologic mixing increases dispersal-related processes in open fluidic systems (Bracken et al., 2013; Meyerhof et al., 2016). Homogeneous selection was the major assembly process impacting BC_FL_ bacterial due to their greater hydrological connectivity. Such higher habitat homogeneity induced by connectivity lead to more similar BC_FL_ than BC_PA_. This notion supports the size-plasticity hypothesis stating that smaller cells reflect a more direct environmental effect (Farjalla et al., 2012). In contrast, for the PA lifestyle, heterogeneous selection was the primary deterministic process, indicating more heterogeneous environmental conditions on particles.

In conclusion, temperature and nitrite were the most important environmental factors for seasonal development of BC_FL_, and temperature and Secchi depth were the key factors driving BC_PA_ dynamics in the Wujiangdu reservoir. These results emphasize the importance of climate-related factors in driving the bacterial community succession in this plateau riverine reservoir. Moreover, nitrate, as the major nitrogen component of in the water column, is related to BC_FL_, e.g., due to nitrifying bacteria converting ammonia to nitrites and nitrates or denitrifying bacteria reducing nitrates, N_2_O and N_2_. In contrast, BC_PA_ was affected by the presence of particles as a substrate for colonization and organic matter resources, whereby the high variations in particle dynamics and composition were driving BC_PA_ composition and their assembly processes.

Our research provides insights into similarities and differences between BC_FL_ and BC_PA_ in a plateau riverine reservoir and evaluates their seasonal dynamics. It improves our understanding of differences in bacterial assembly mechanism and environmental interactions between BC_FL_ and BC_PA._ these results could serve as a basis to predict further changes of bacterial community under the effects of global warming and eutrophication.

## Data availability statement

The data presented in the study are deposited in the Sequence Read Archive (SRA) repository, accession number PRJNA1136858, the link is http://www.ncbi.nlm.nih.gov/bioproject/1136858.

## Author contributions

YY: Funding acquisition, Investigation, Writing – original draft, Conceptualization, Formal analysis. CC: Data curation, Methodology, Visualization, Writing – original draft. KY: Resources, Software, Validation, Writing – review & editing, Supervision. H-PG: Writing – review & editing, Funding acquisition, Project administration, Supervision.
