# Supplementary material for: Seasonal dynamics of free-living (FL) and particle-attached (PA) bacterial communities in a plateau reservoir
Source: Front Microbiol. 2024 Jul 19;15:1428701. doi: 10.3389/fmicb.2024.1428701 (PMC11295932; doi:10.3389/fmicb.2024.1428701)
Supplement: Supplementary file 1 [file Data_Sheet_1.DOCX]

**Figures**


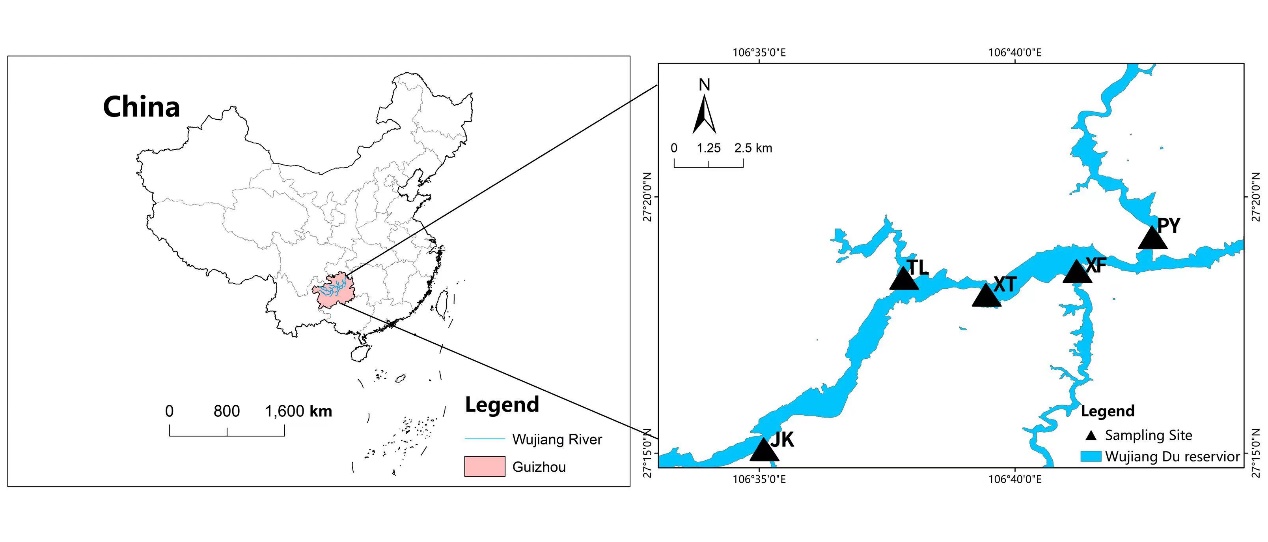


Fig. 1 Sampling locations in the Wujiangdu reservoir, China (5 sites: JK, TL, XT, XF and PY)

**
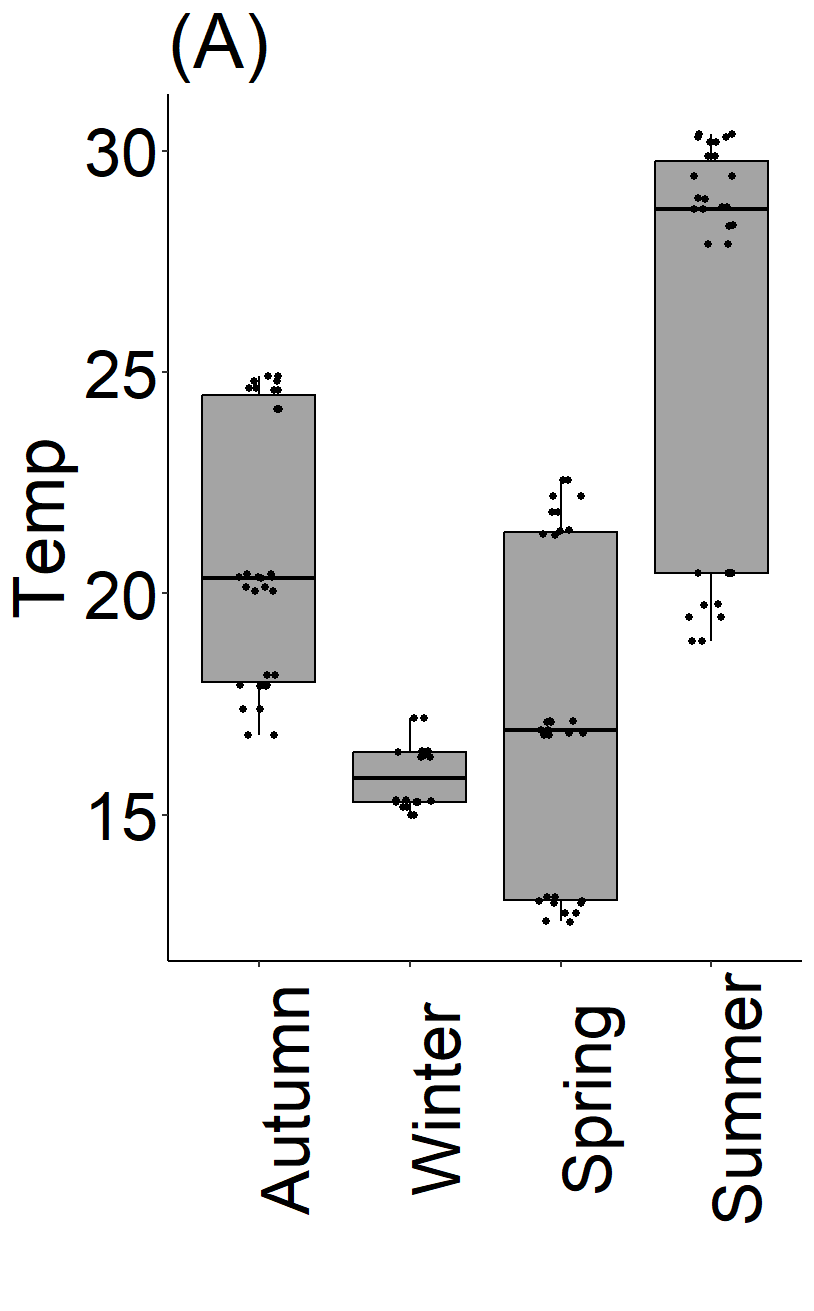

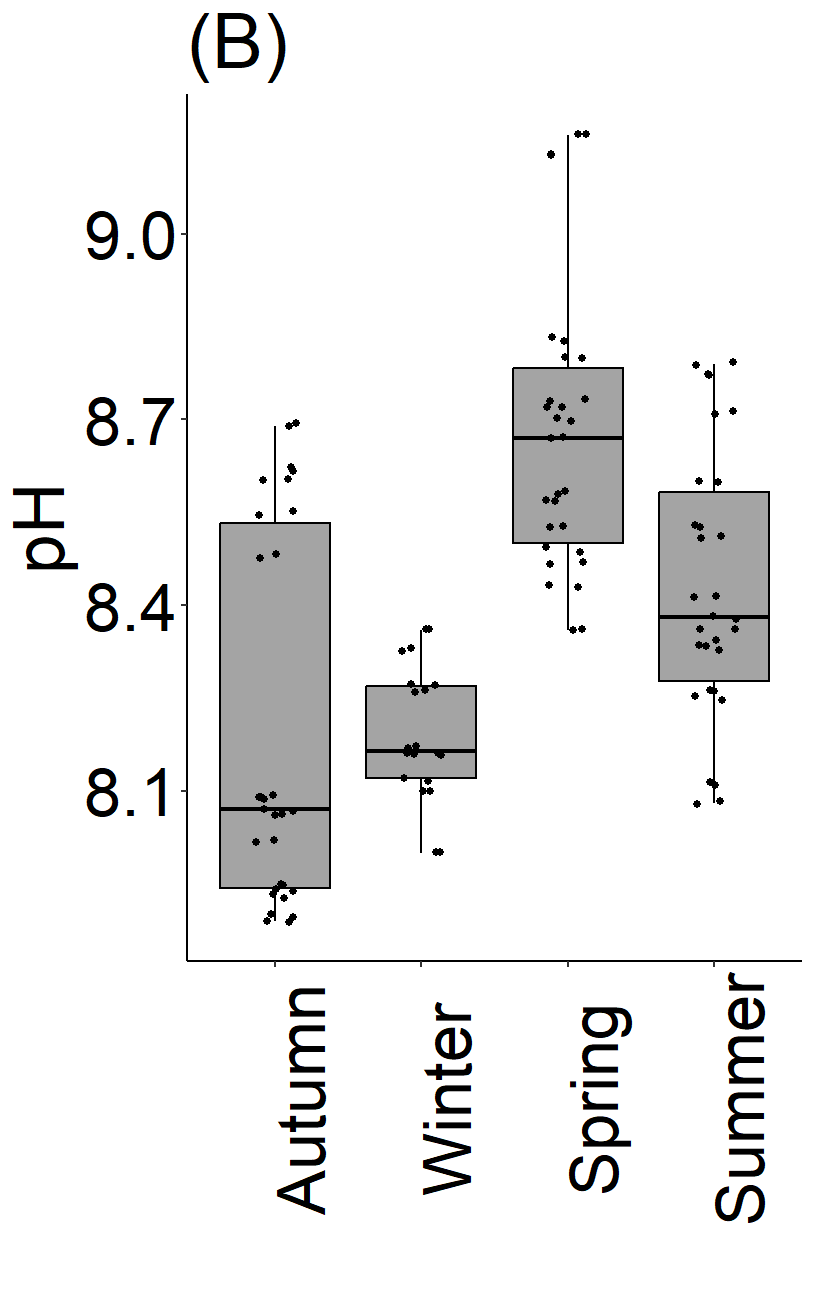

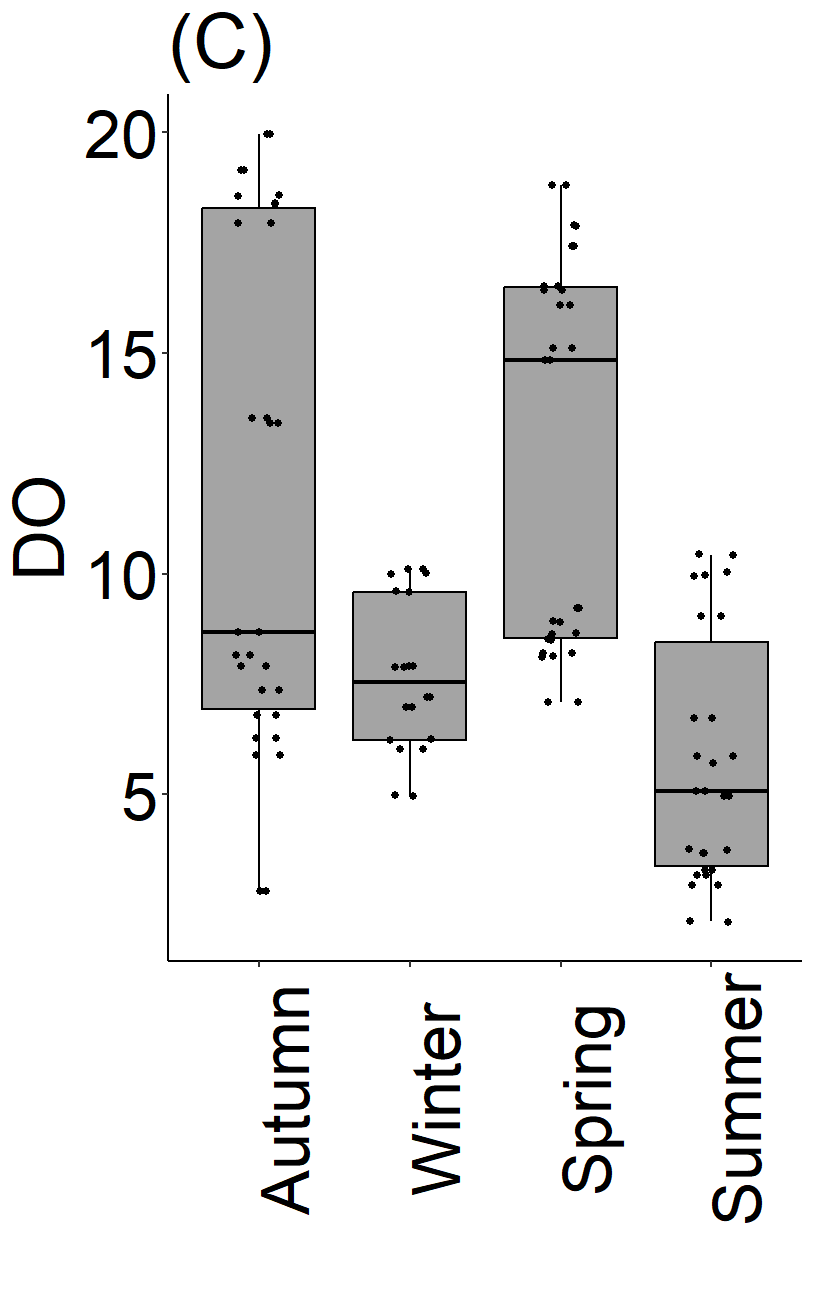
**

**
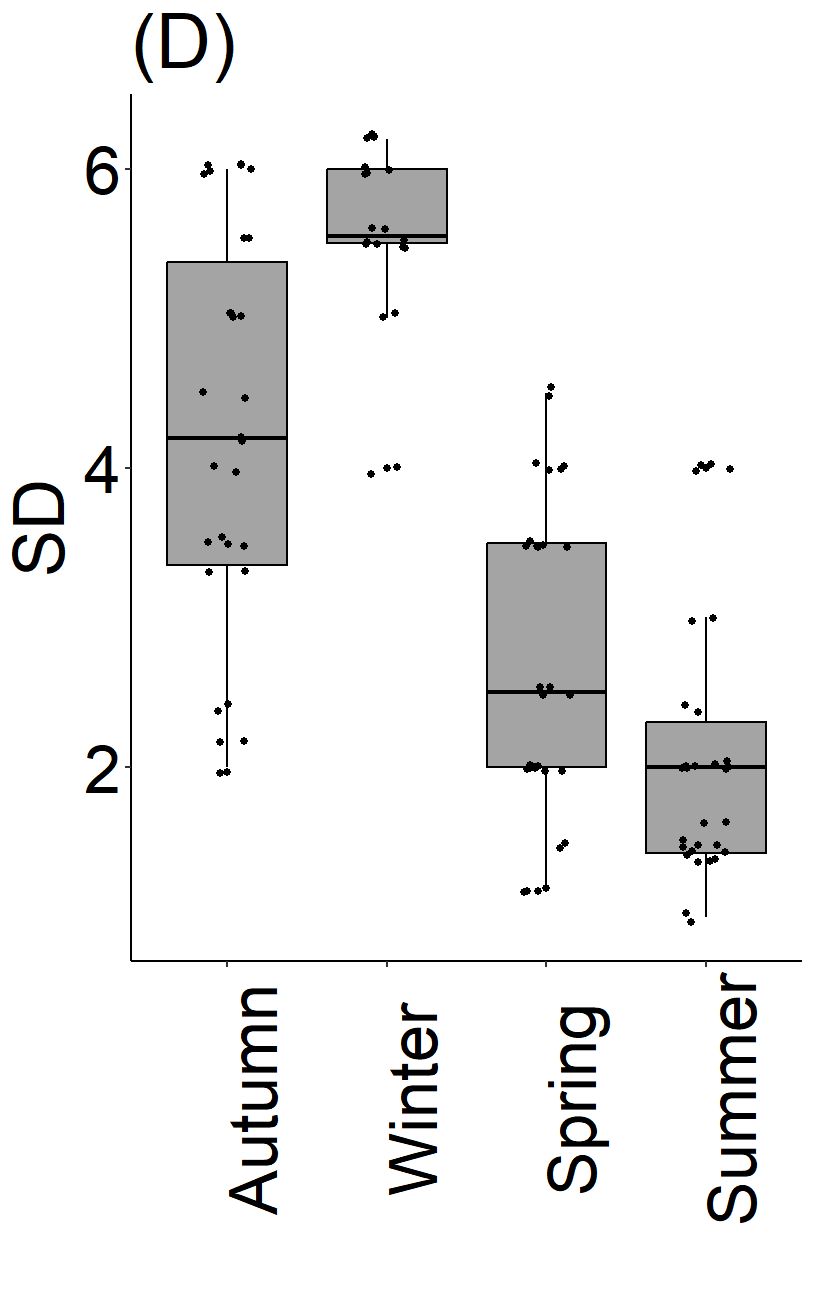

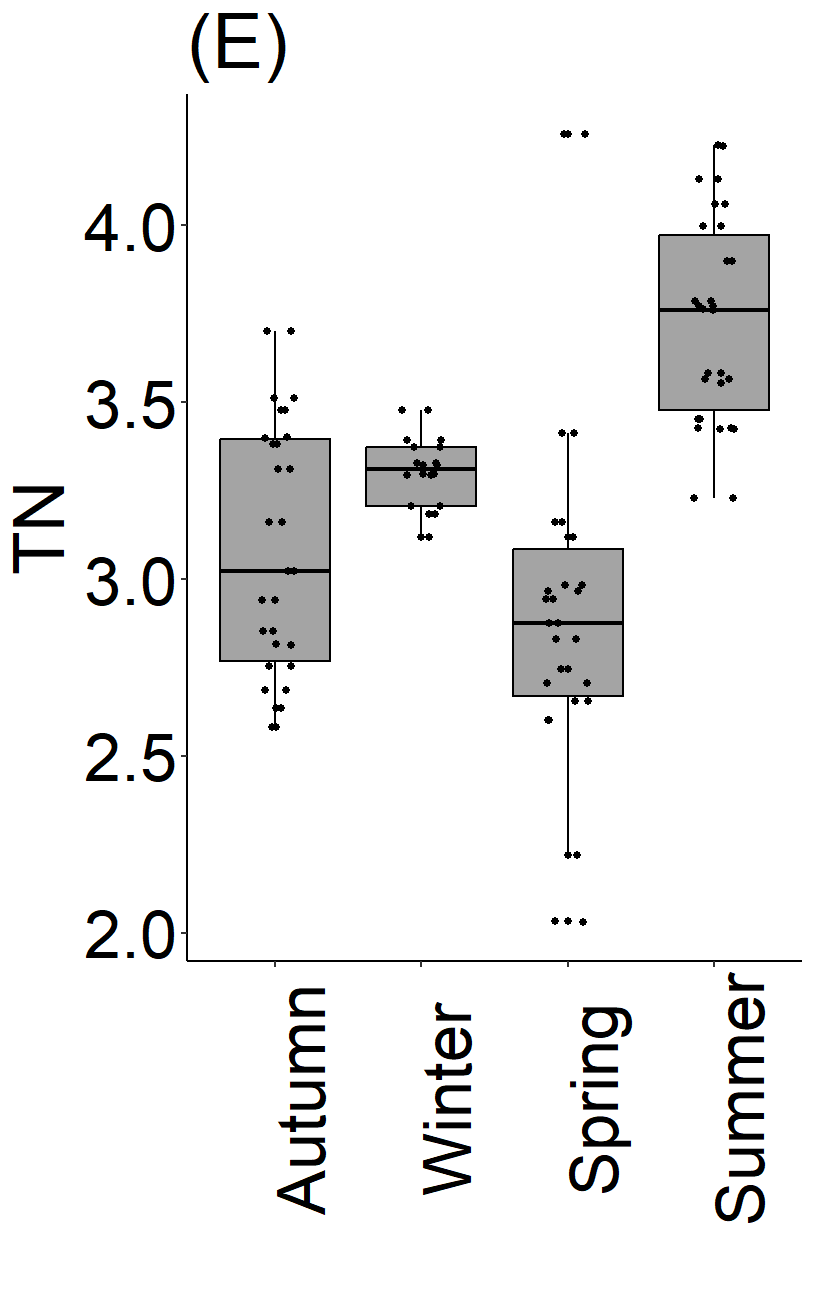

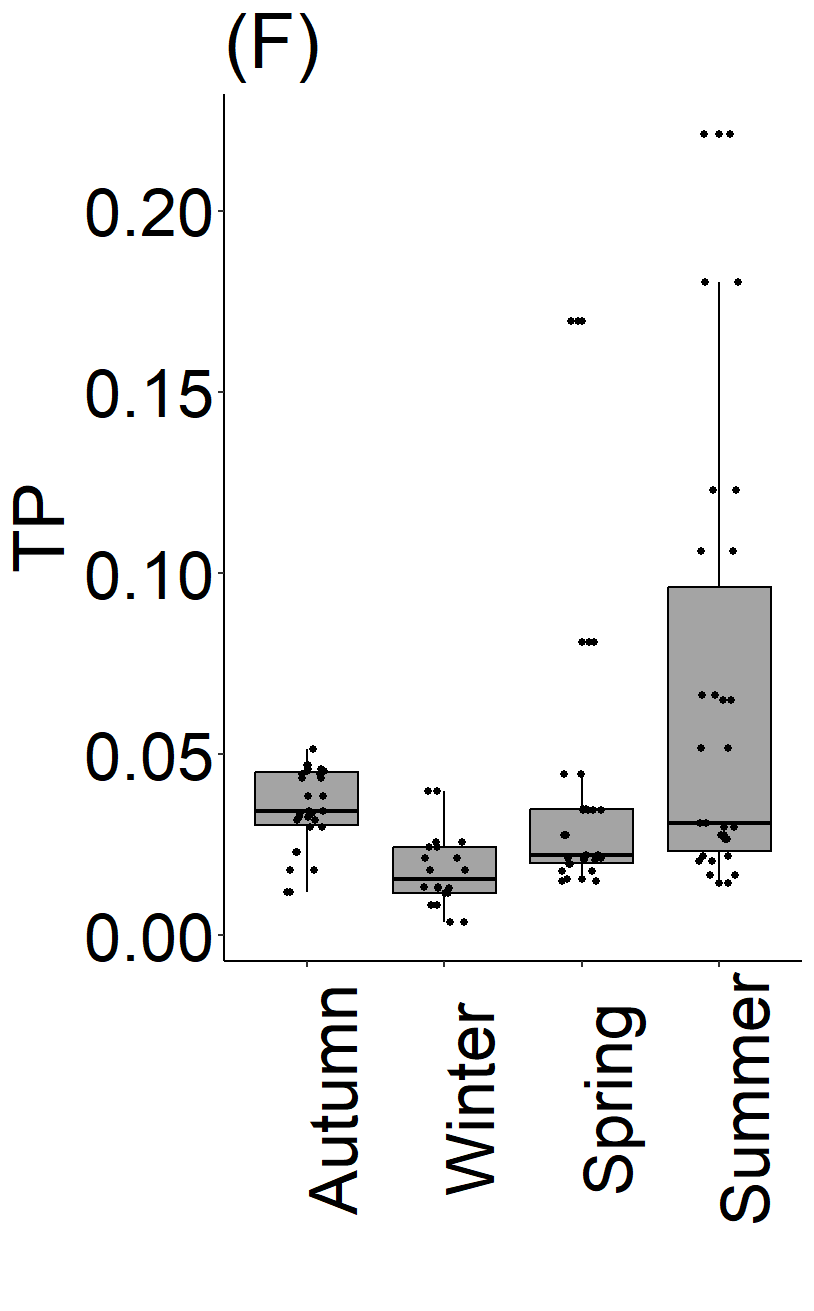
**

**
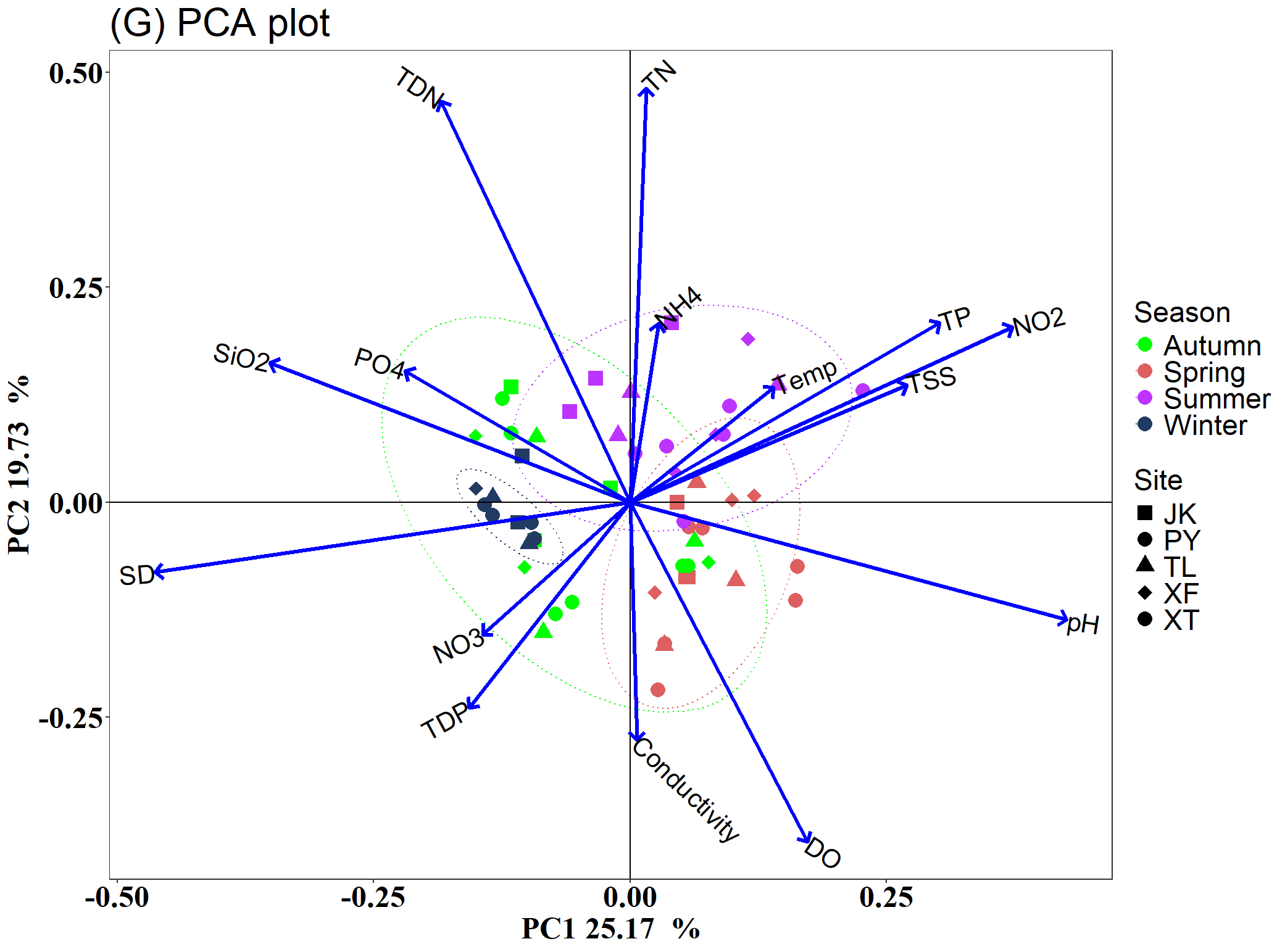
**

Fig. 2 Seasonal variations of environmental variables (a) Temp: water temperature; (b) TSS: total suspended solid; (c) SD: Secchi depth; (d) pH; (e) TN: total nitrogen; (f) TP: total phosphorus); and (g) PCA on all measured environmental factors.

**
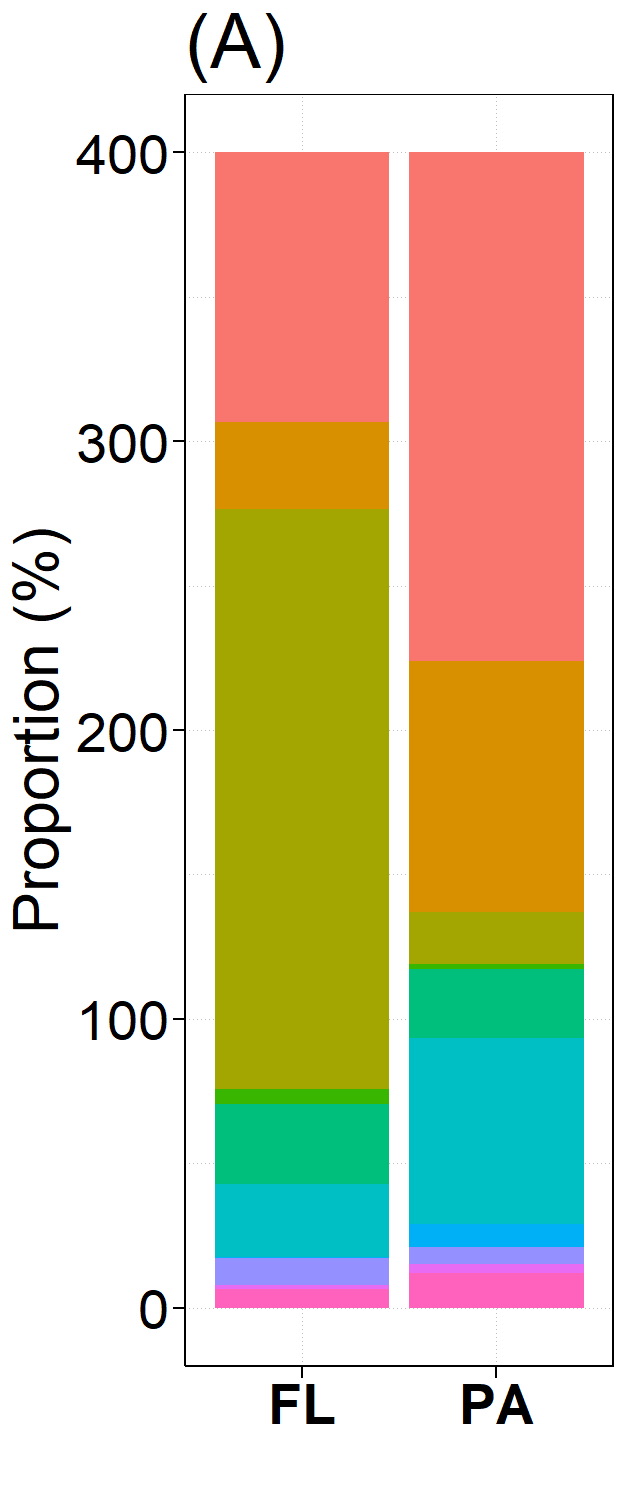

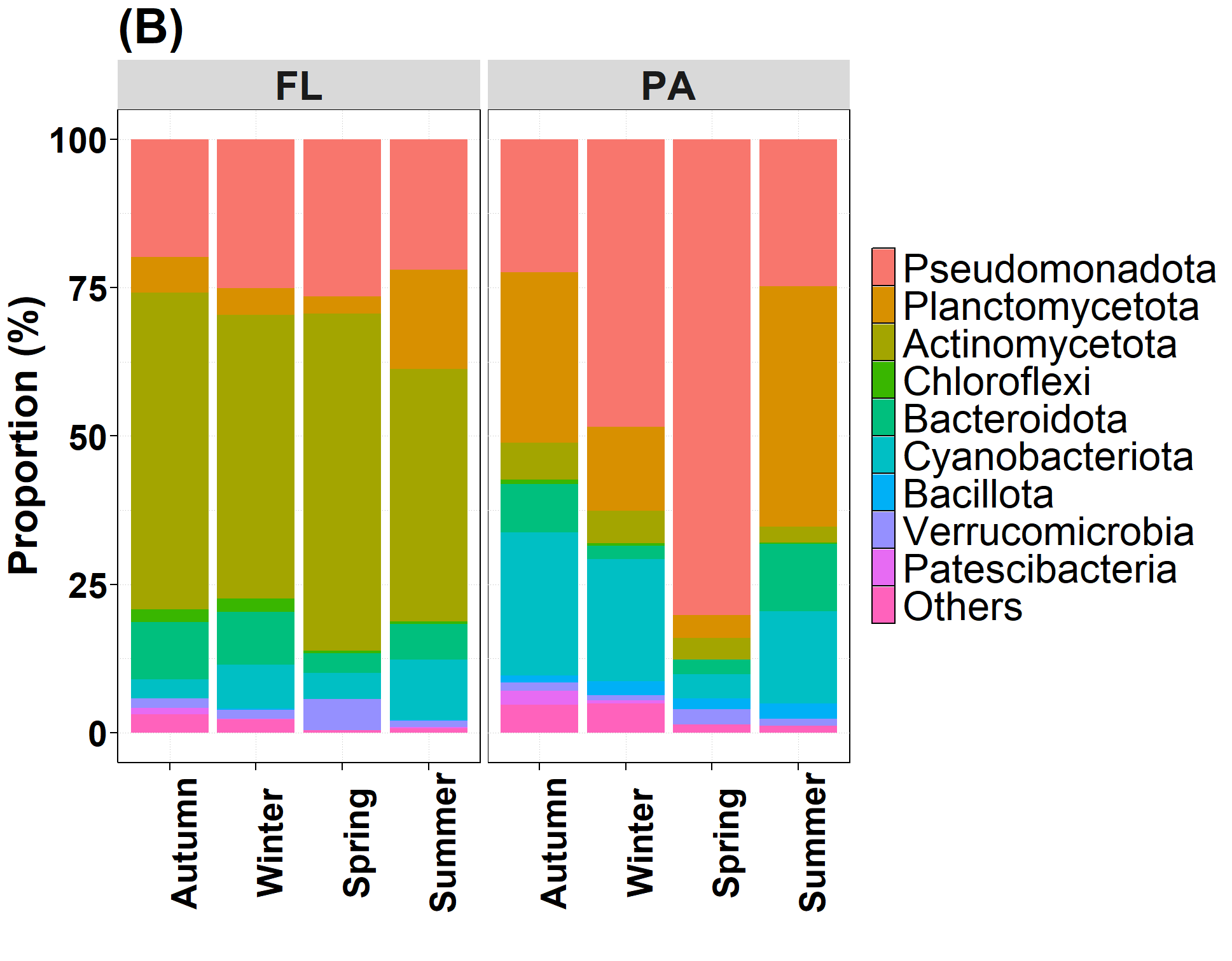
**

Fig. 3 Variations in relative abundance of (a) the top 10 phyla of FL and PA bacterial communities, and (b) across seasons in the Wujiangdu reservoir.

**
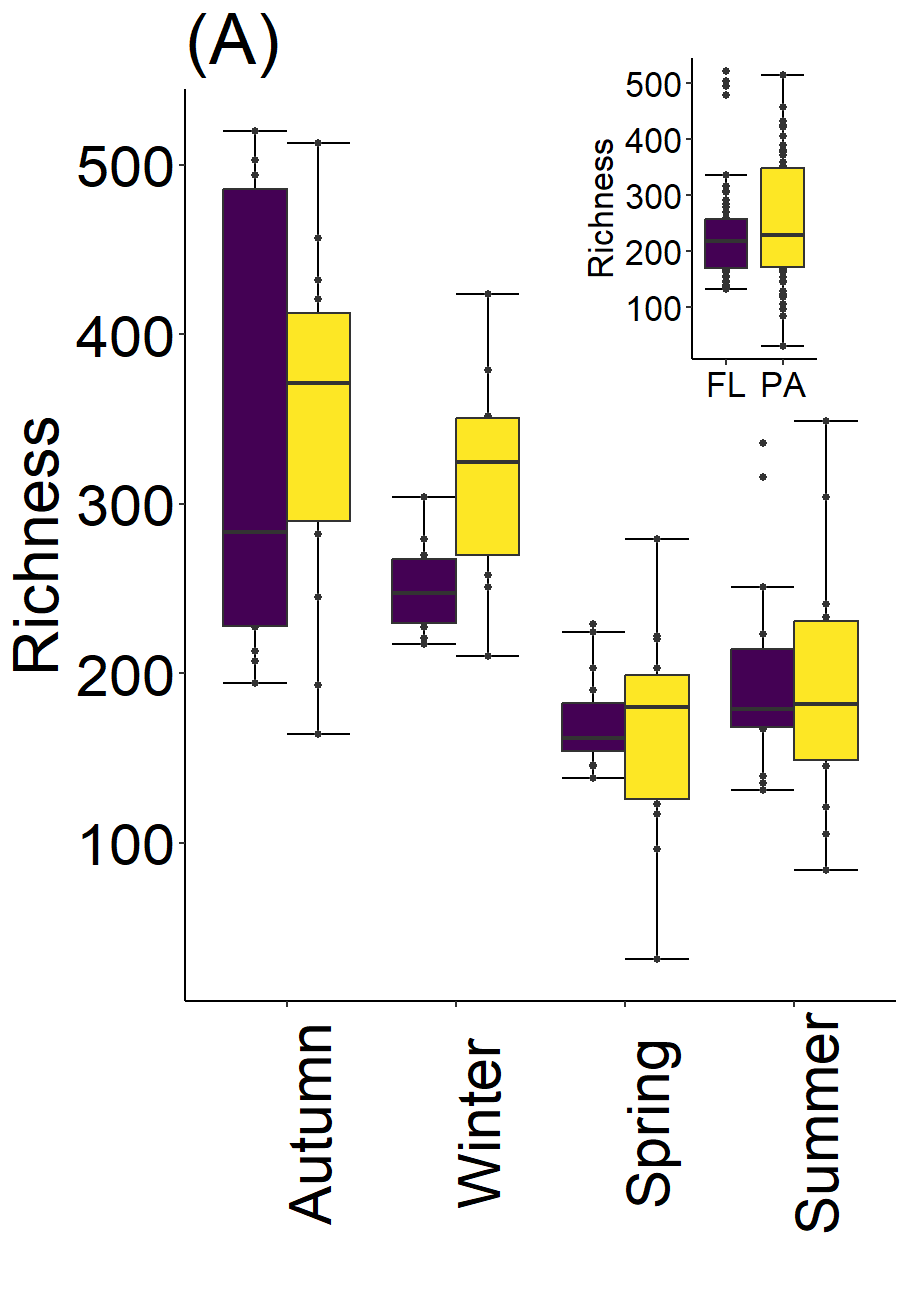

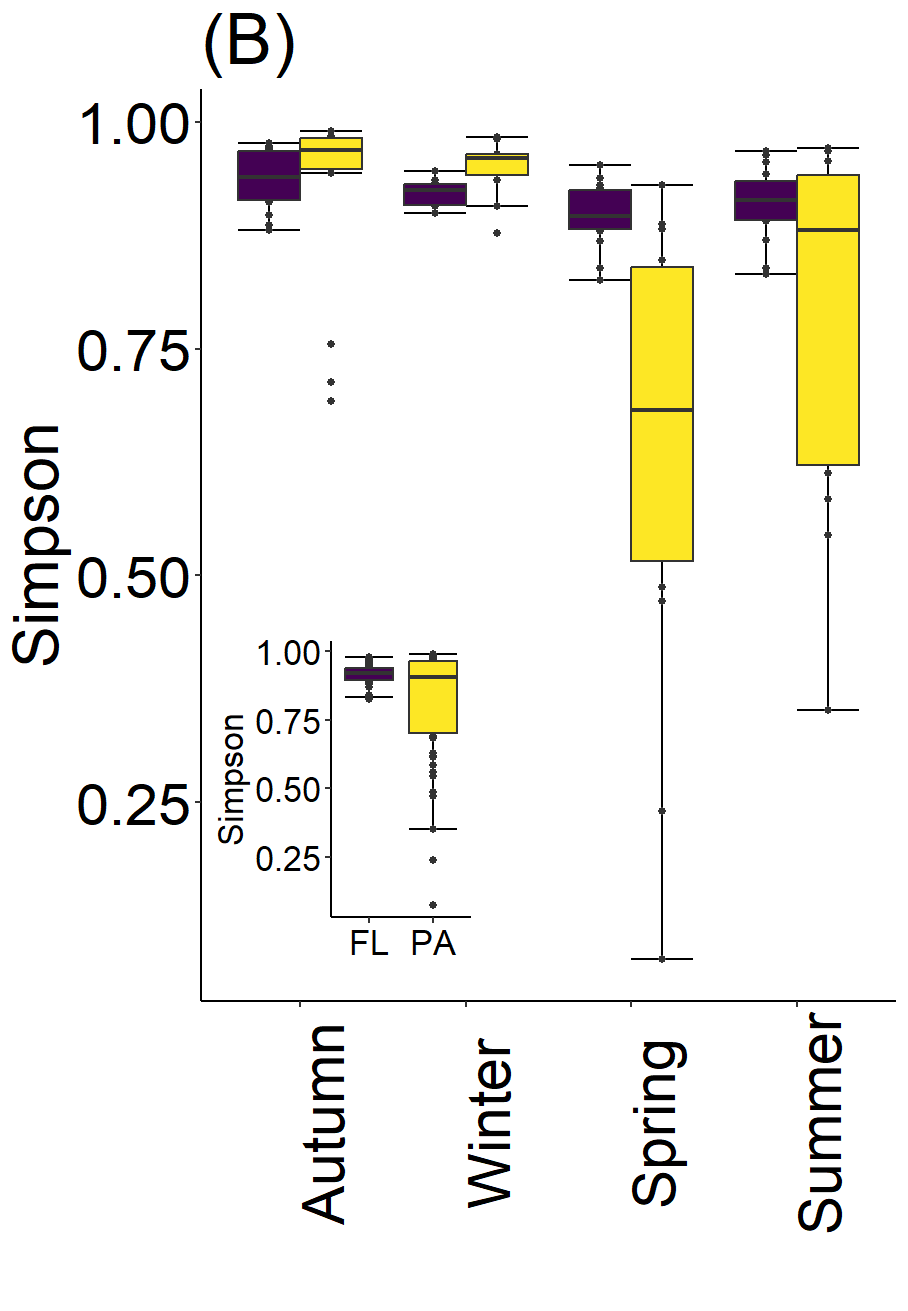

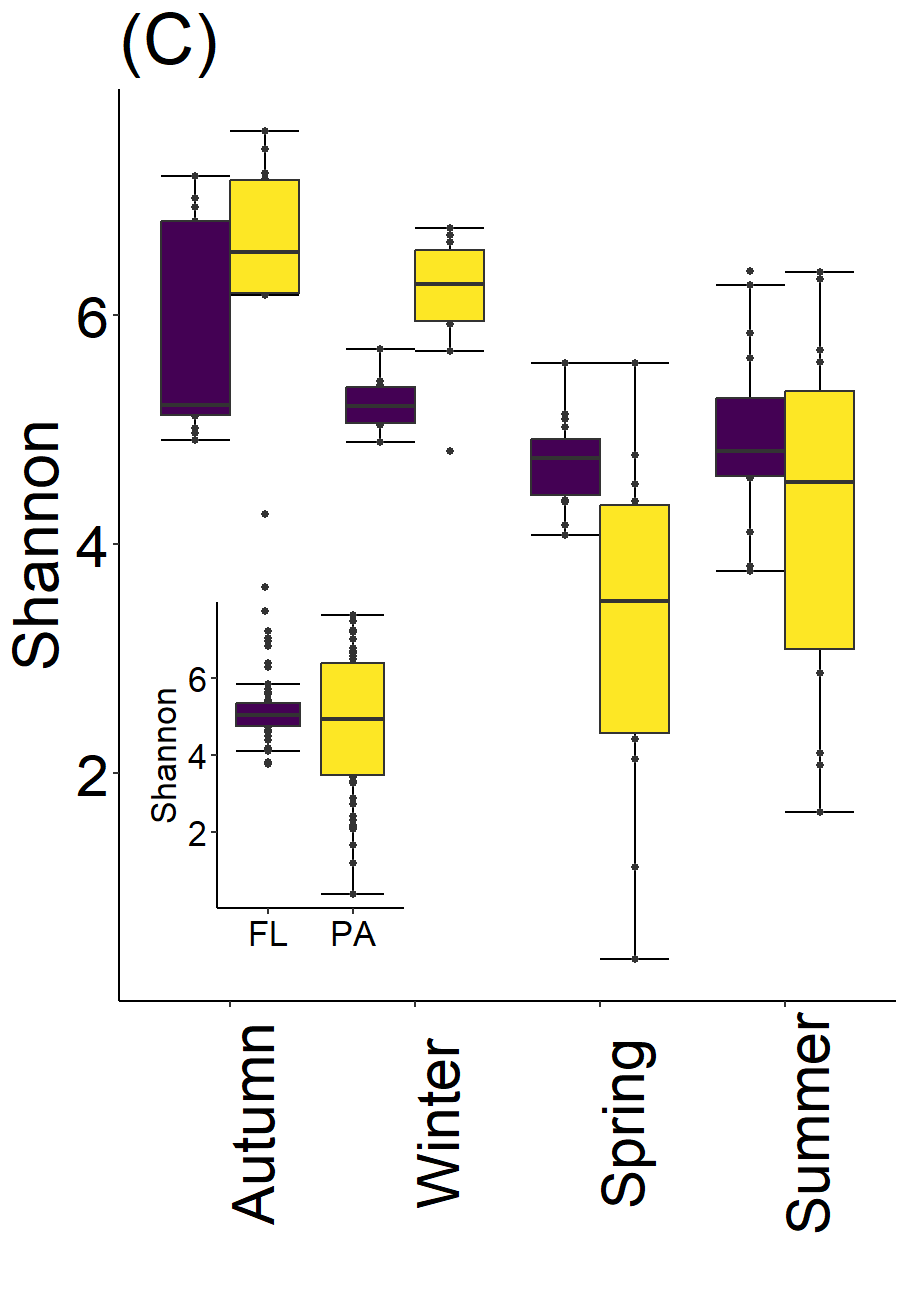

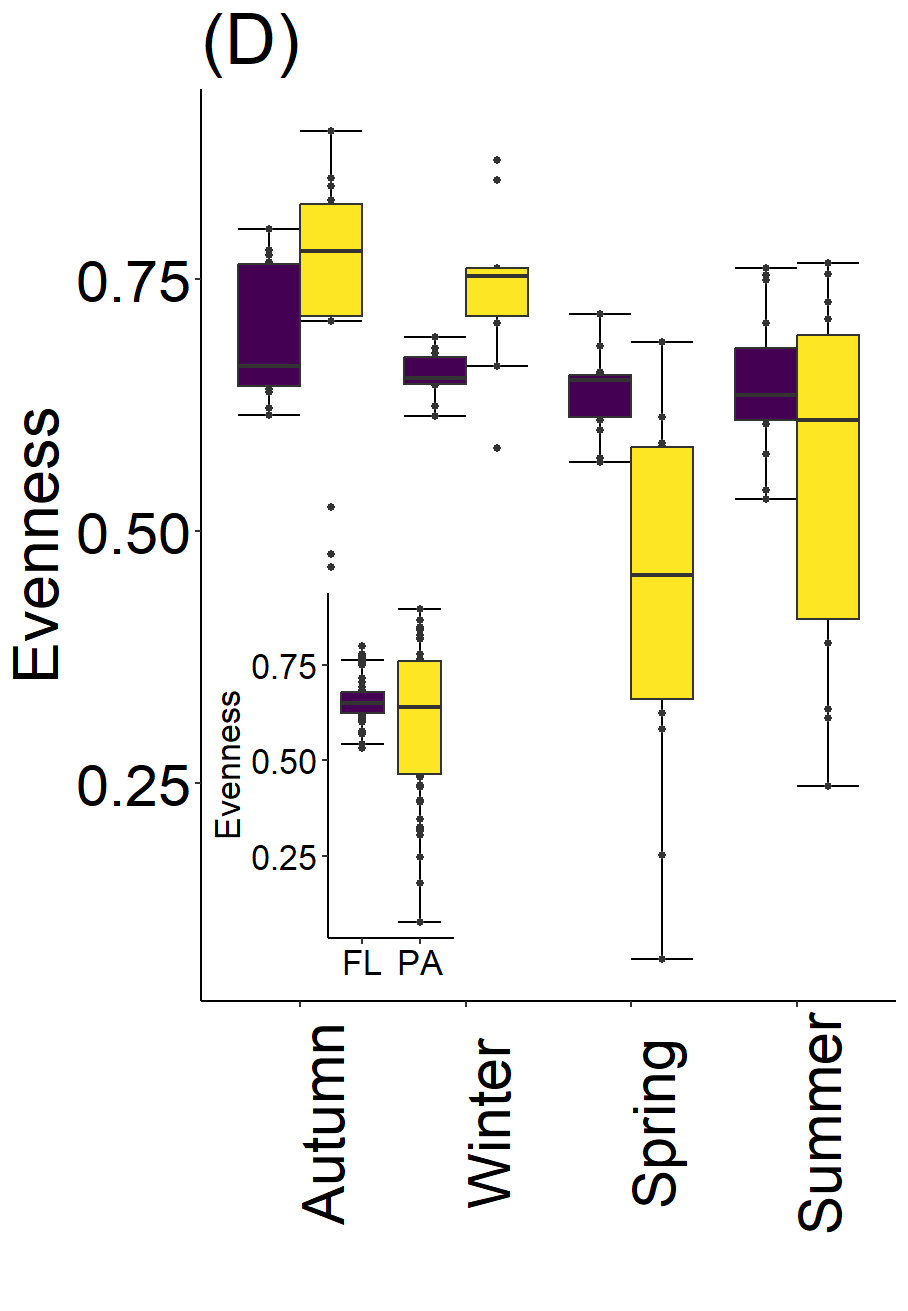

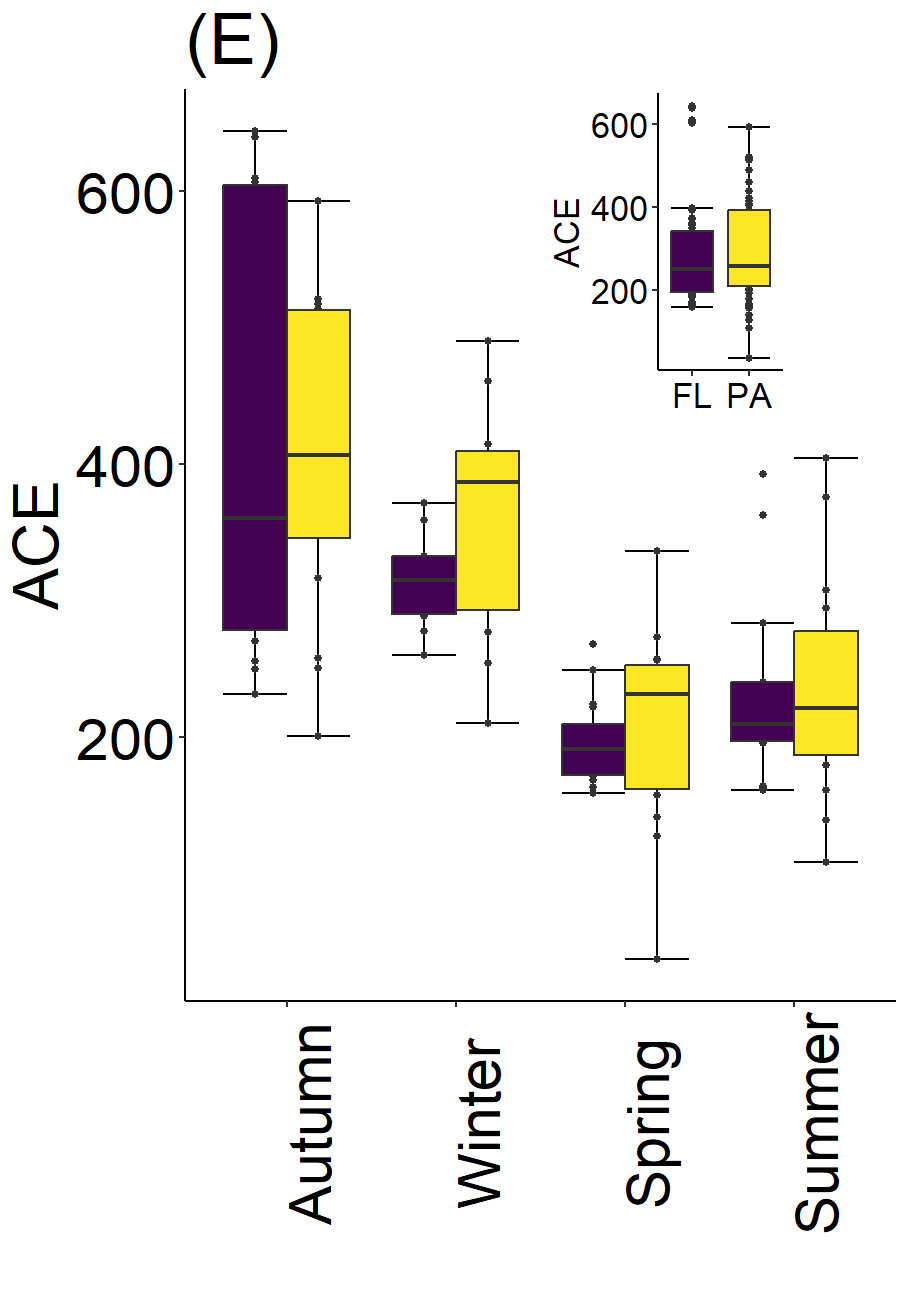

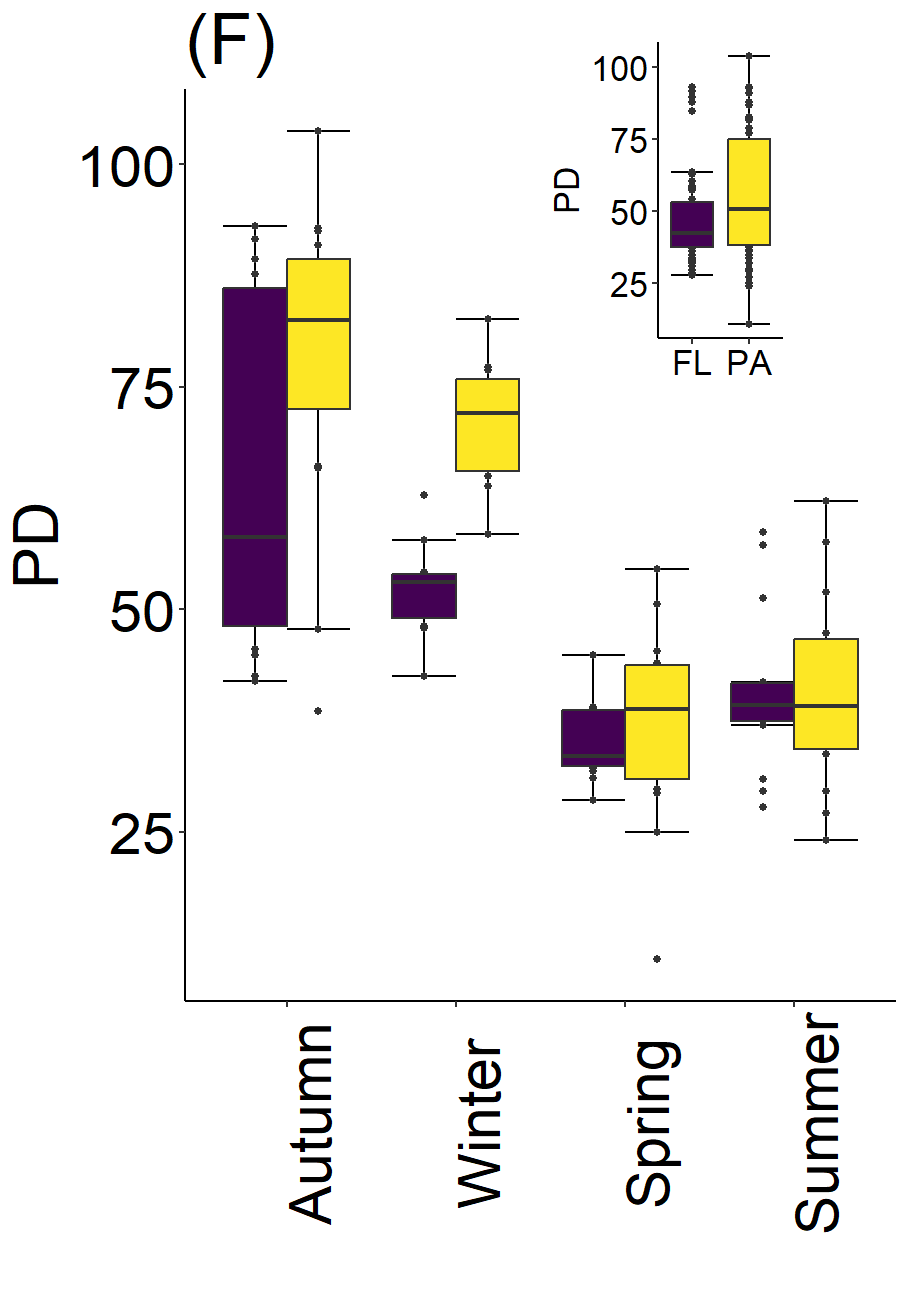
**

Fig. 4 Seasonal variations of α-diversity of BC_FL_ and BC_PA_ in the Wujiangdu Reservoir (a) ASV richness; (b) Simpson; (c) Shannon; (d) Pielou’s Evenness; (e) ACE; (f) PD.

**
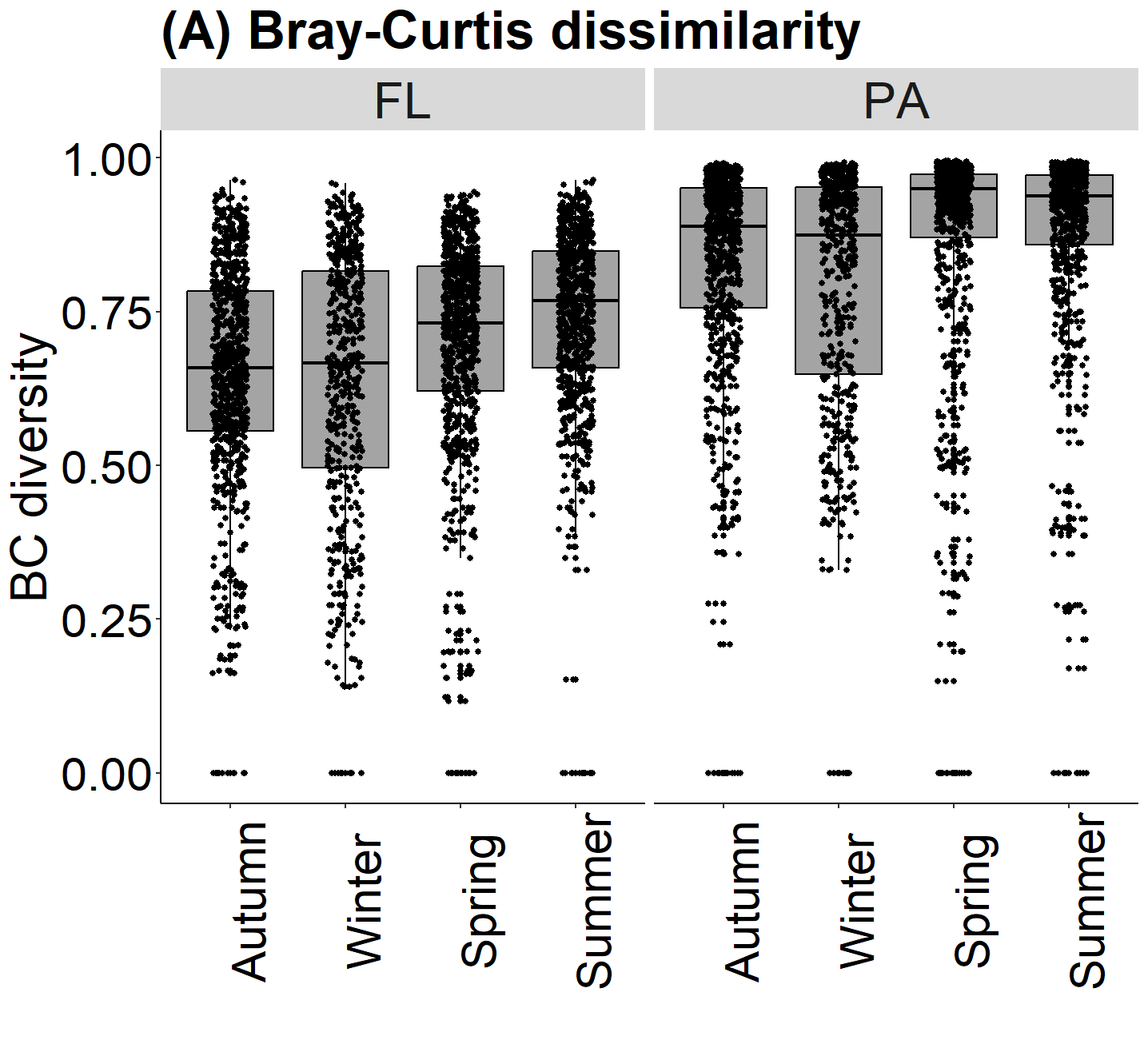

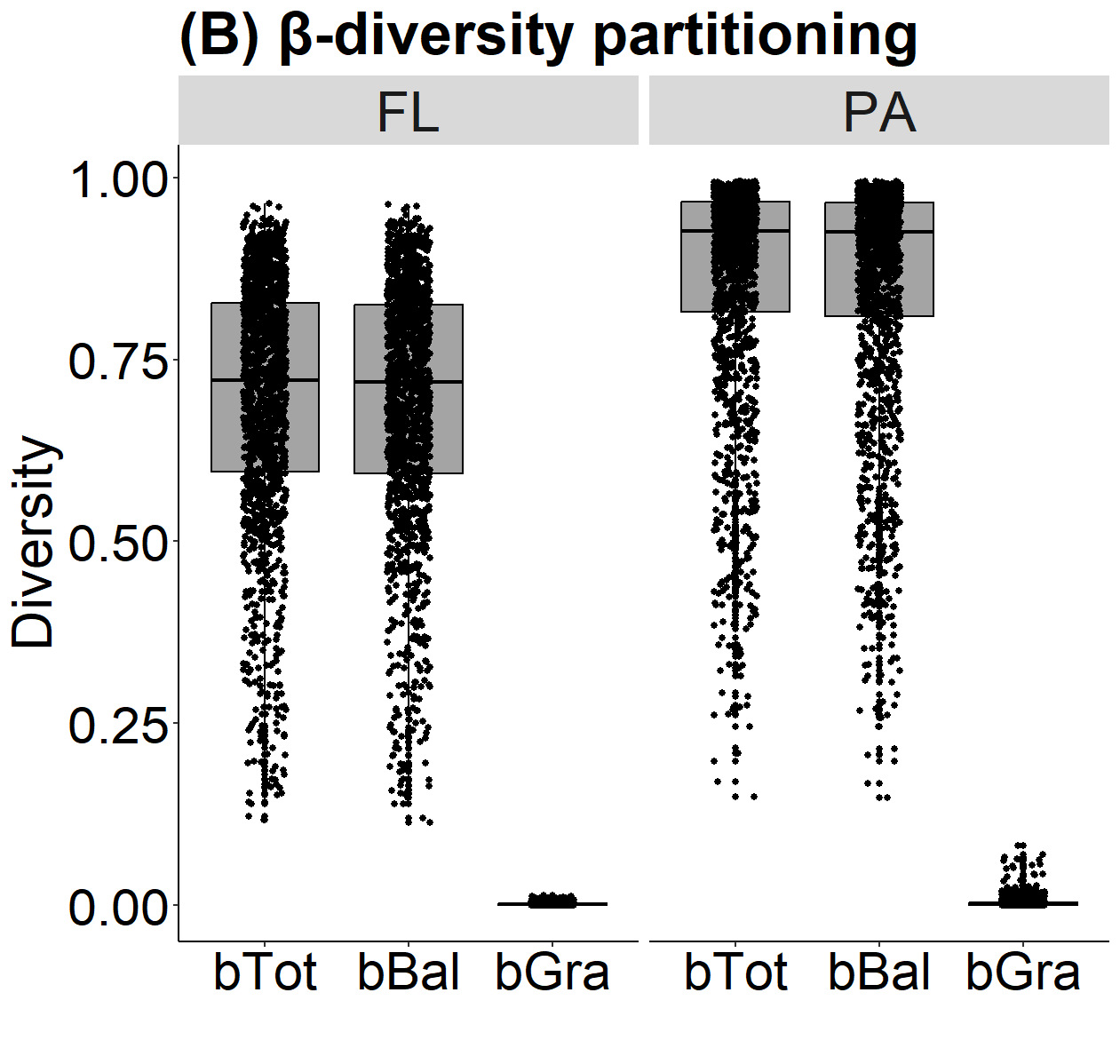
**

**
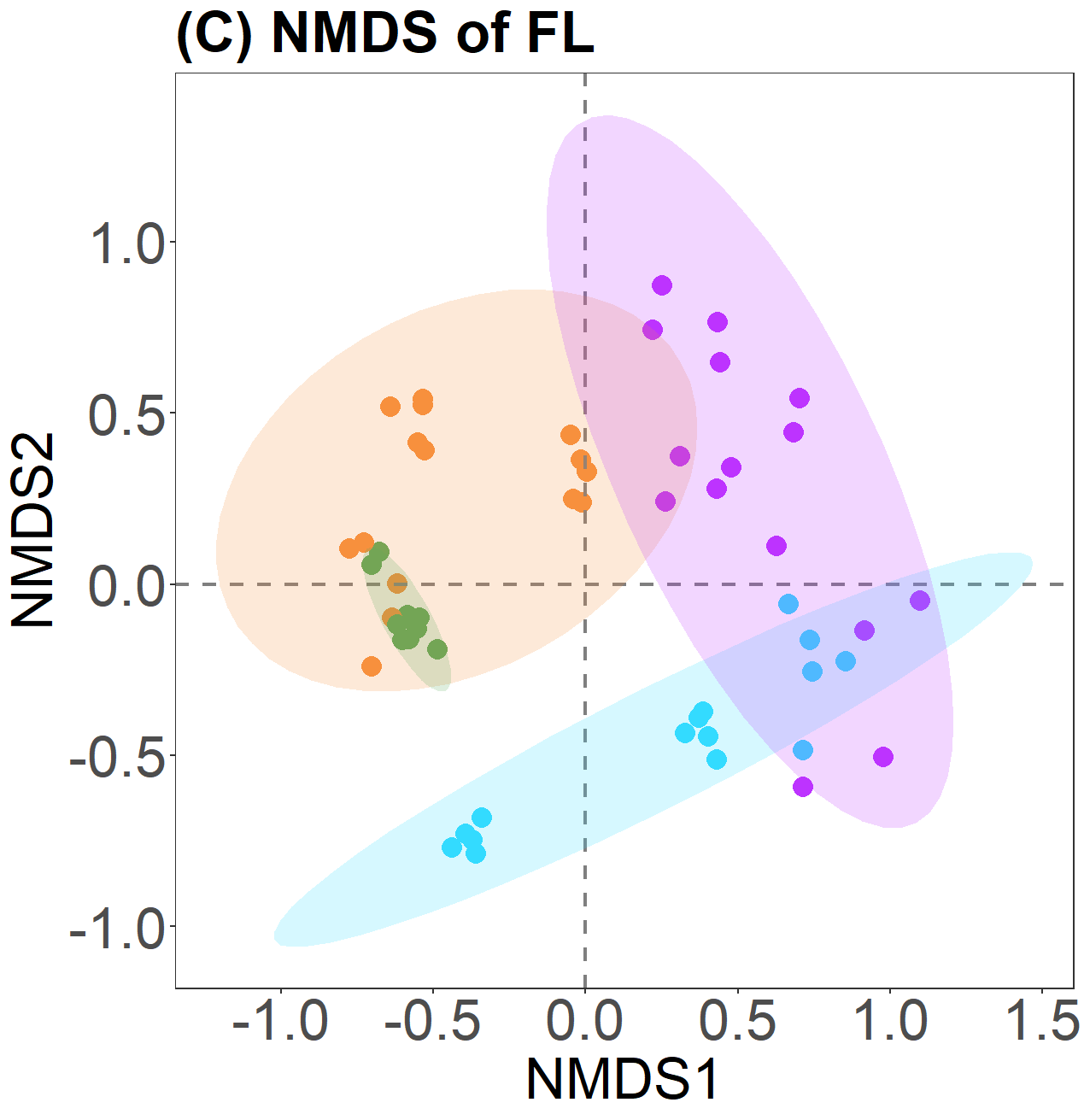

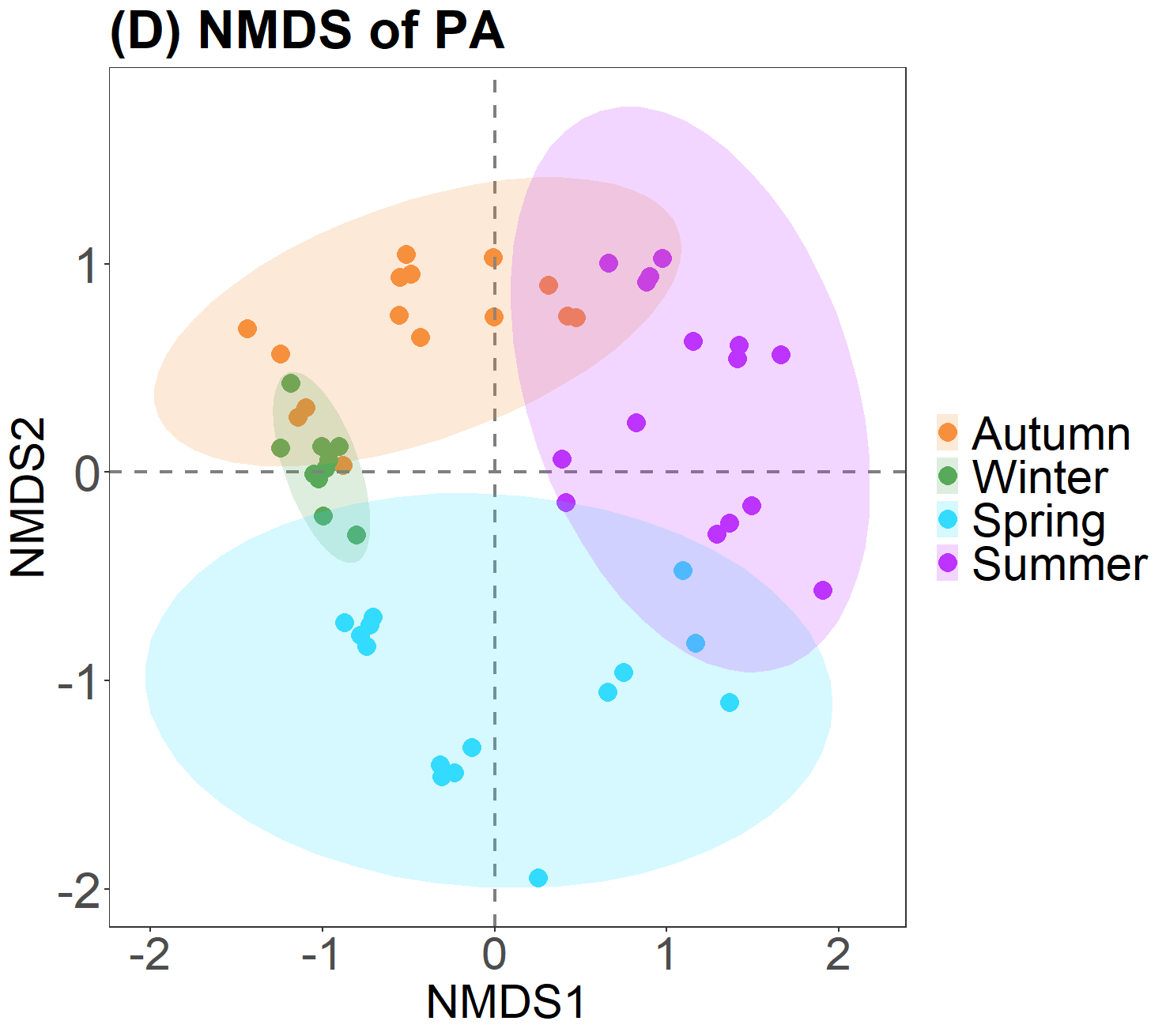
**

Fig. 5 Seasonal variation in Bray-Curtis dissimilarity of BC_FL_ (a) and BC_PA_ (b); β-diversity partitioning for BC_FL_ and BC_PA_ (c); and NMDS plot of BC_FL_ (d) and BC_PA_ (e).


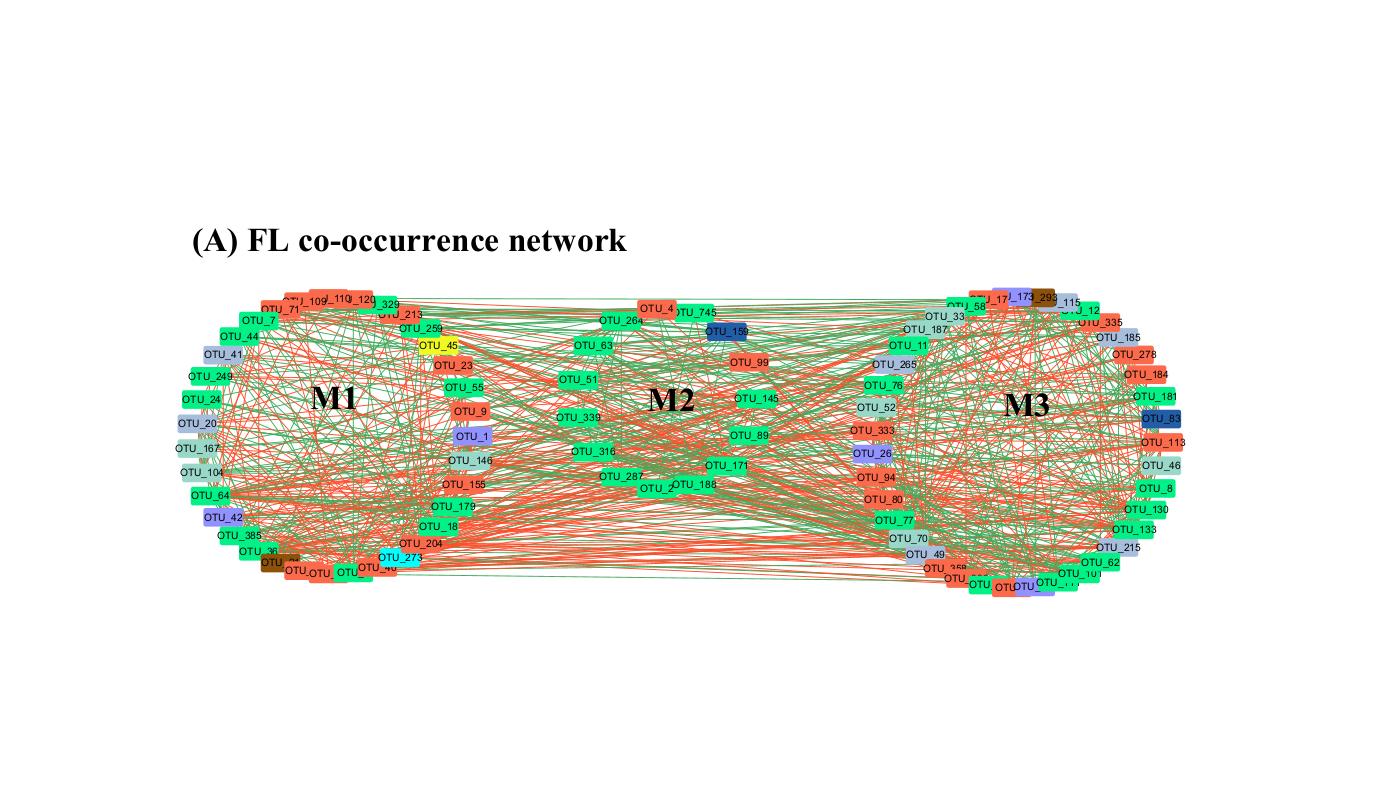

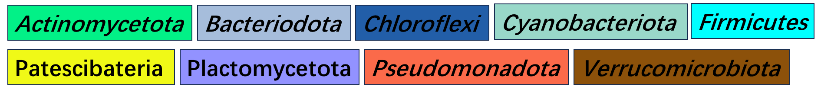

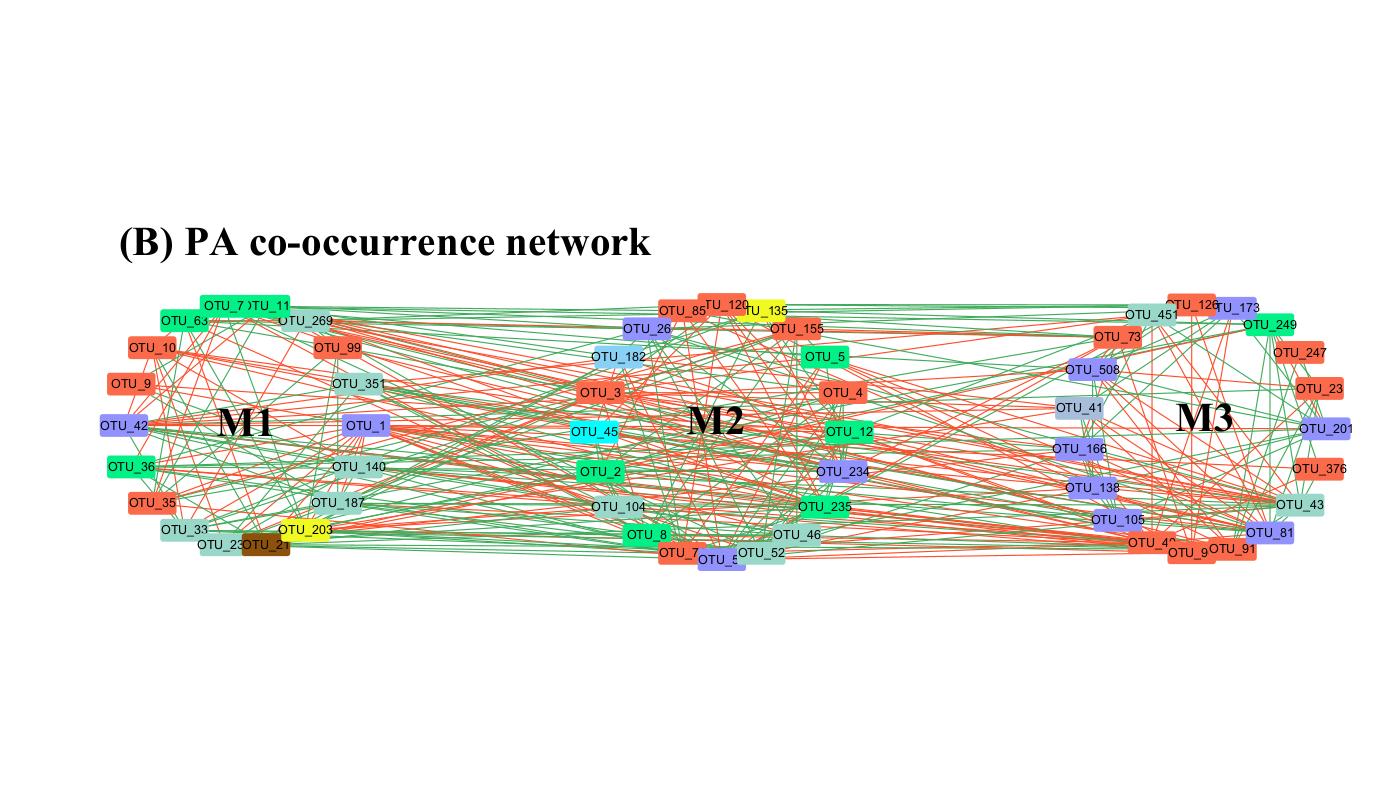

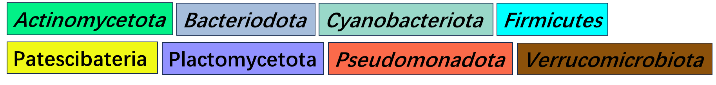


**
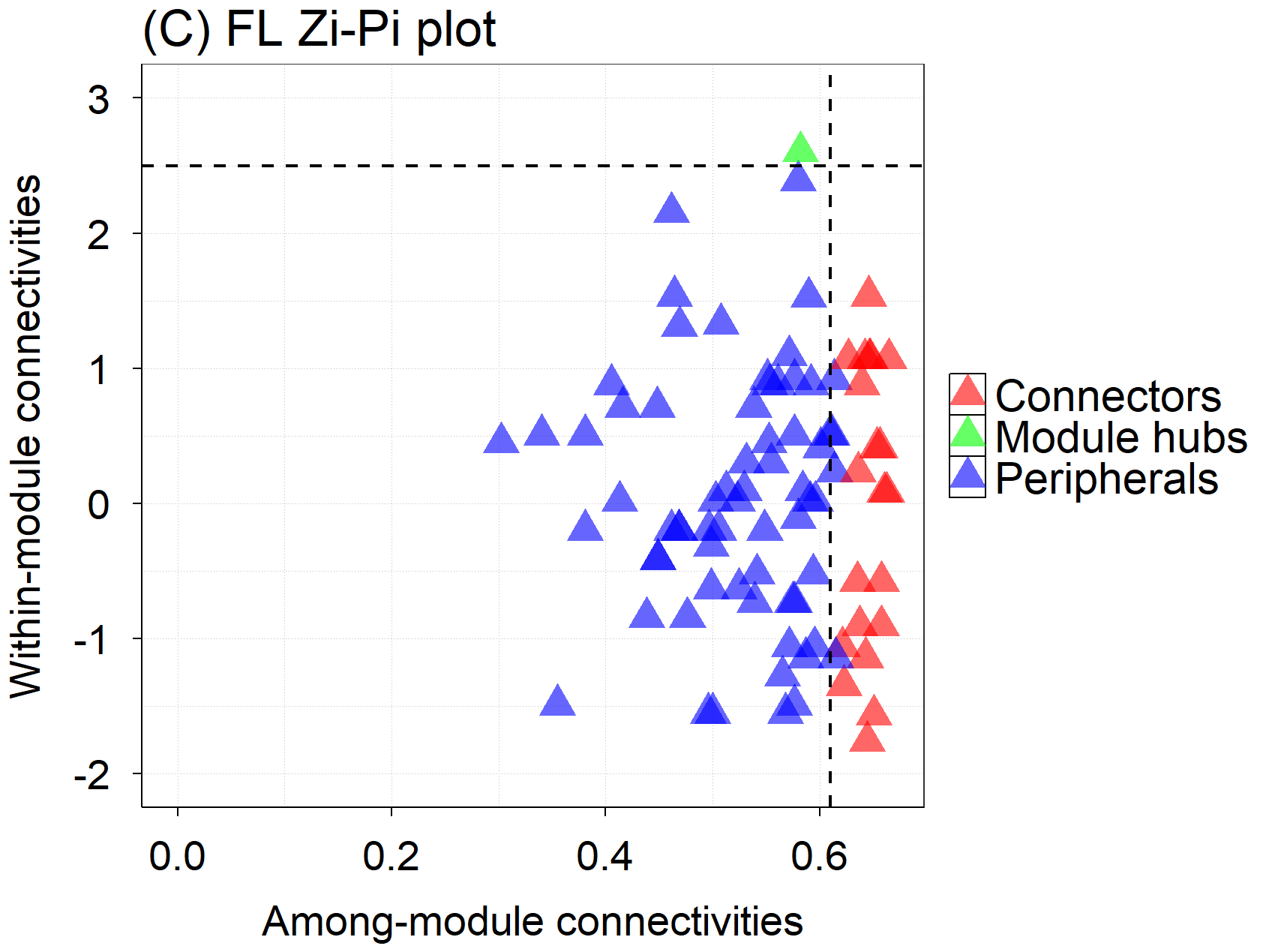

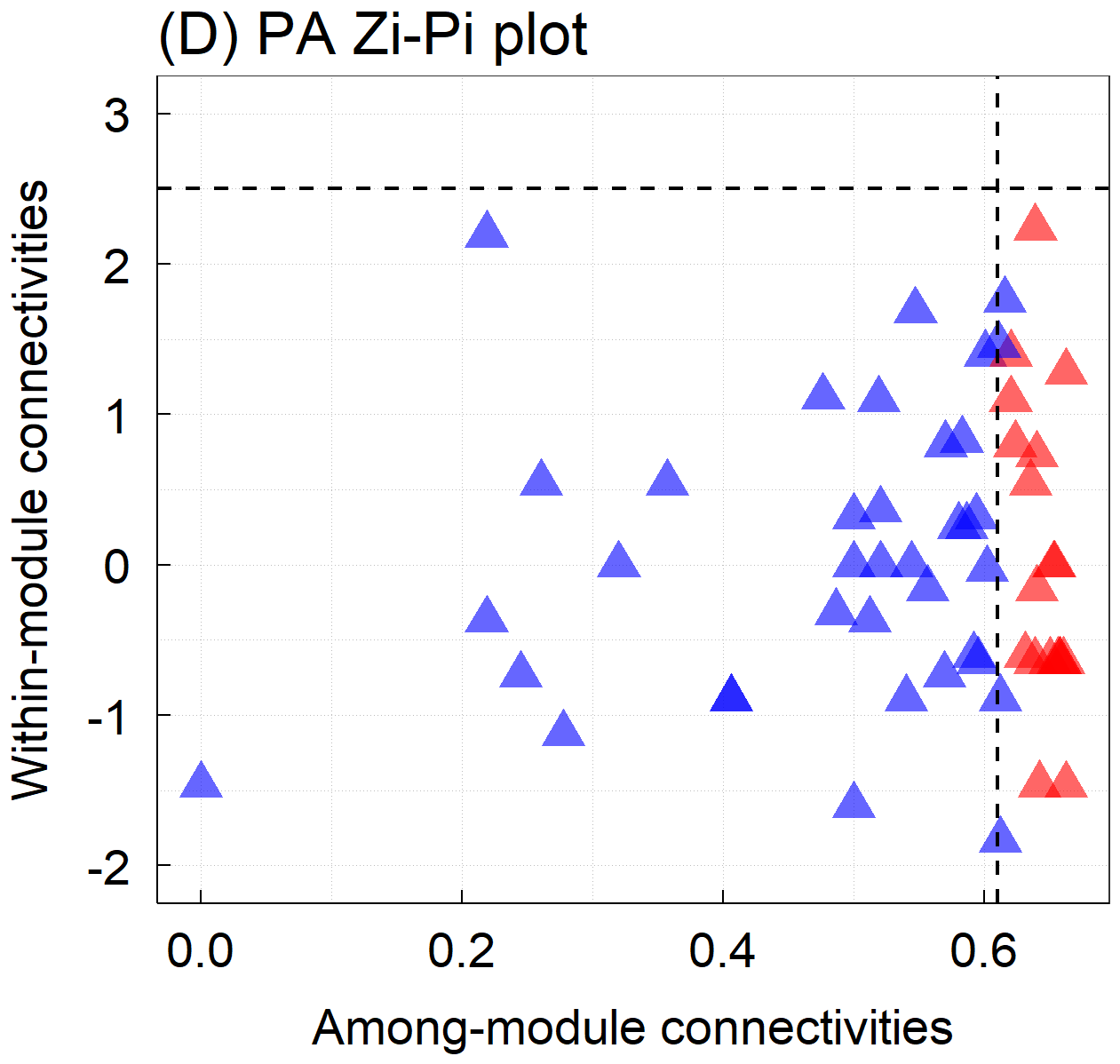
**

Fig. 6 Network analysis of BC_FL_ (a) and BC_PA_ (b); Zi-Pi plot of ASVs in BC_FL_ (c) and BC_PA_ (d) networks (the color of nodes refers to the phylum of the OTU; green edges: positive correlation; red edges: negative correlation; M1-M3 refers to modules in each network; Zi: within-module connectivity; Pi: among-module connectivity)

**
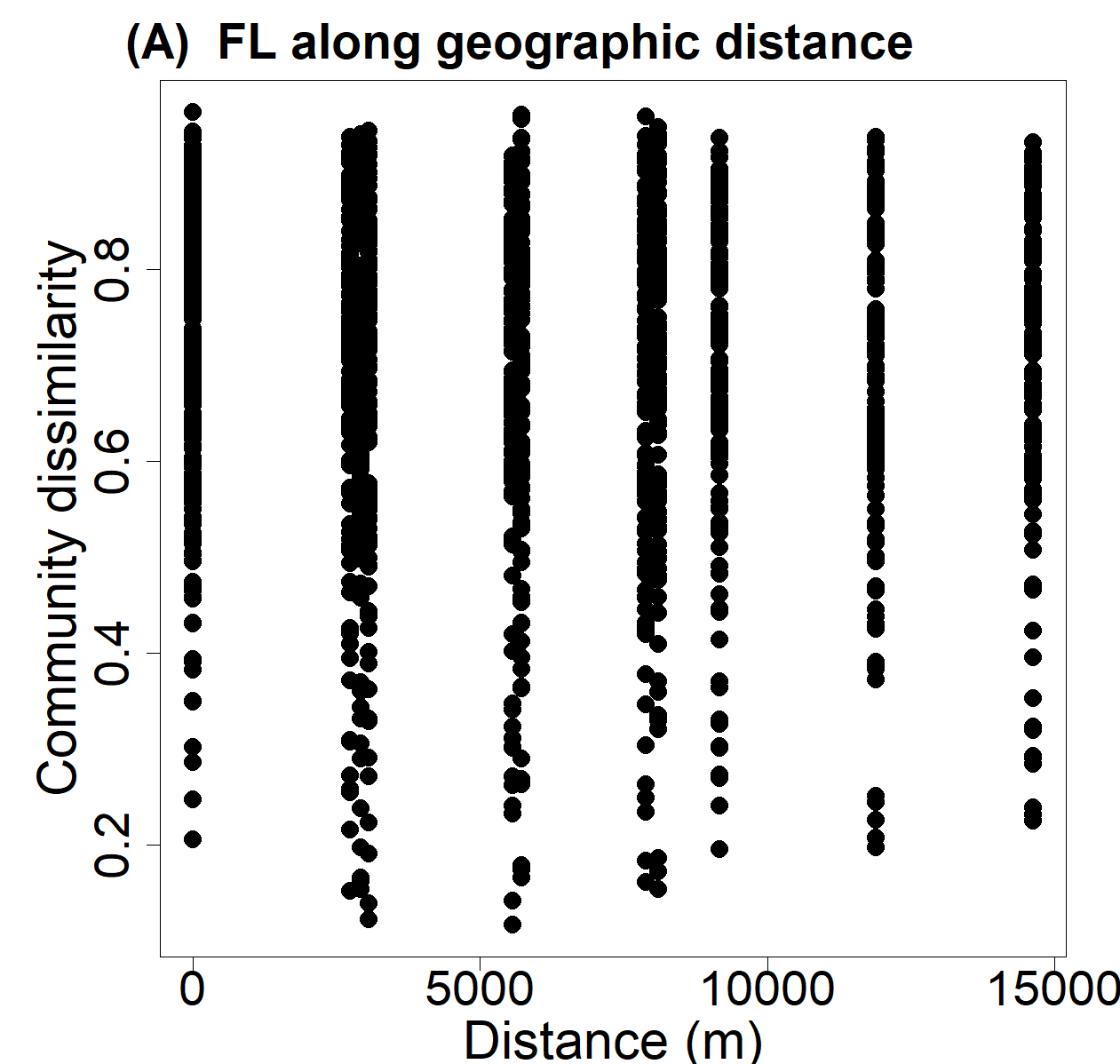

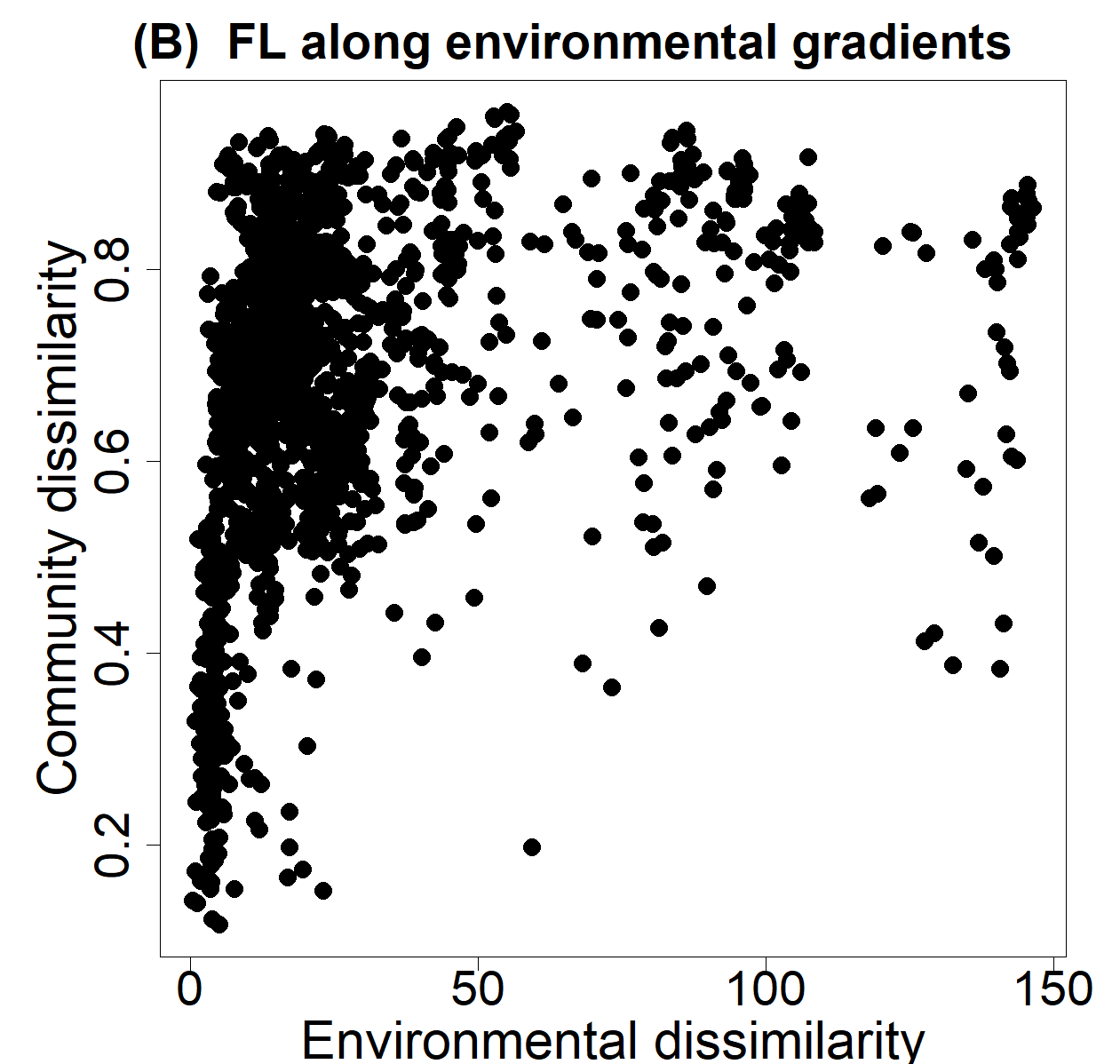
**

**
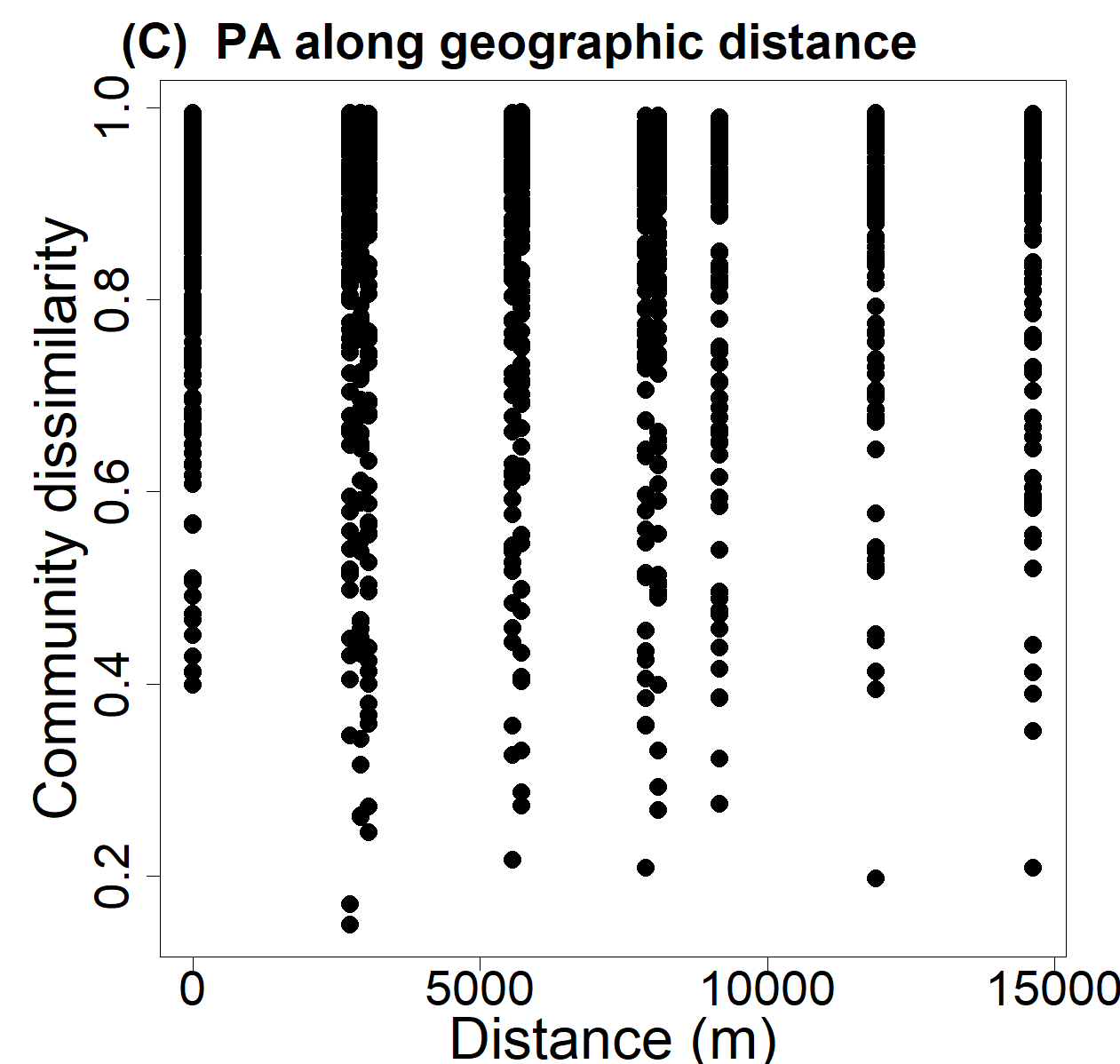

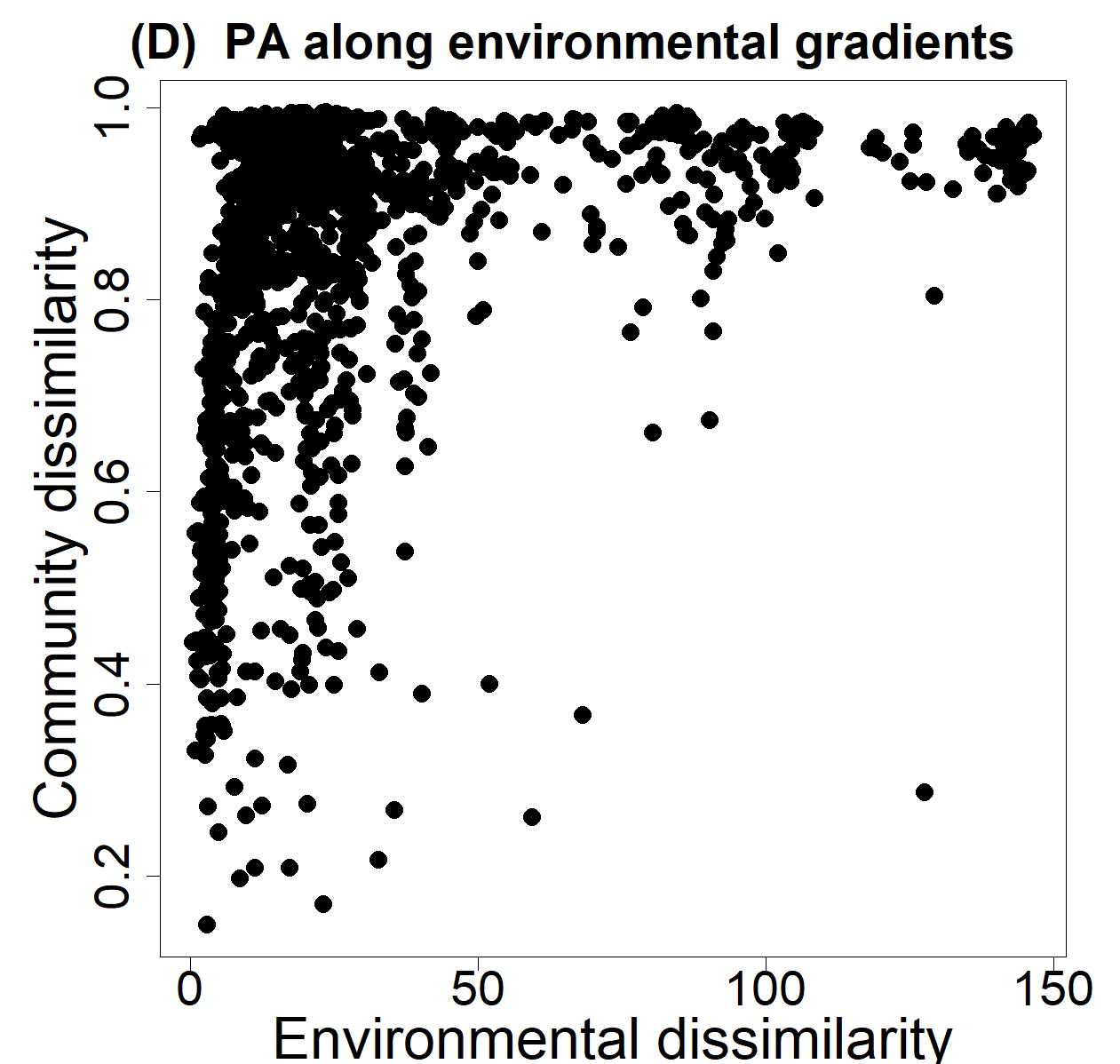
**

**
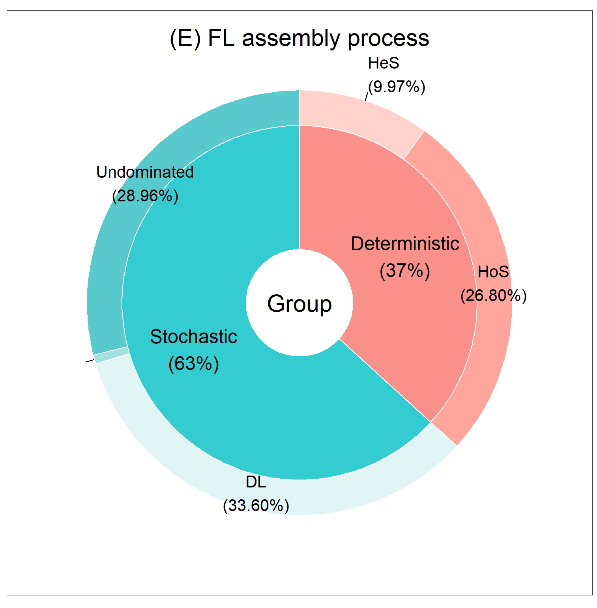

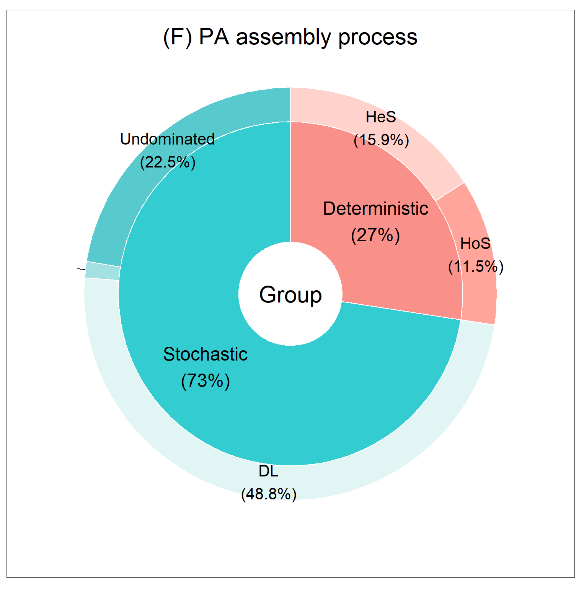
**

Fig. 7 Relationship between community similarity (Bray- Curtis distance from the ASV table) of BC_FL_ and BC_PA_ related to (a and c) geographic distance and (b and d) environmental similarity; Bacterial community assembly processes for (e) BC_FL_ and (f) BC_PA_. DL: dispersal limitation, HeS and HoS: heterogeneous and homogeneous selection, respectively.

**
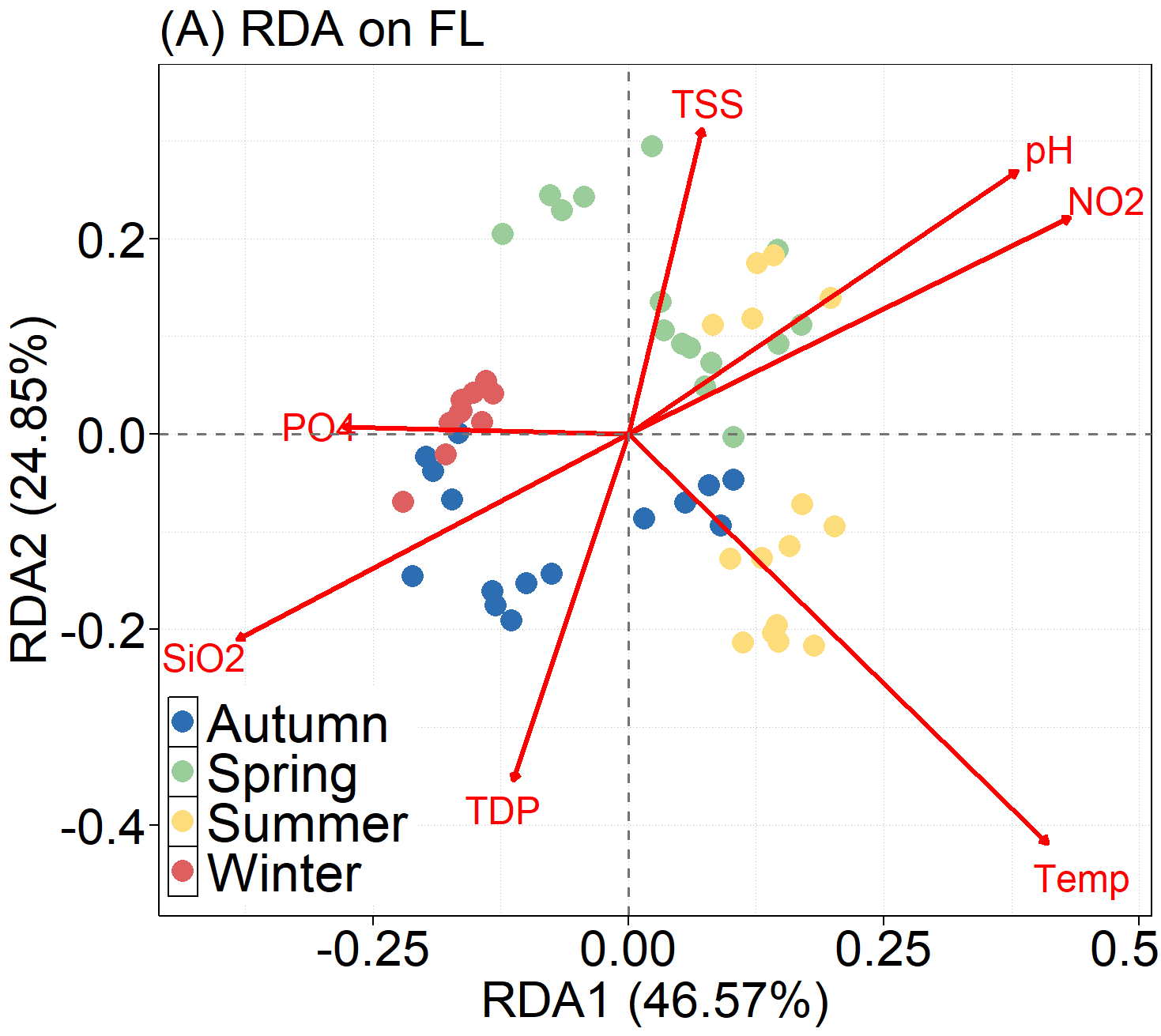

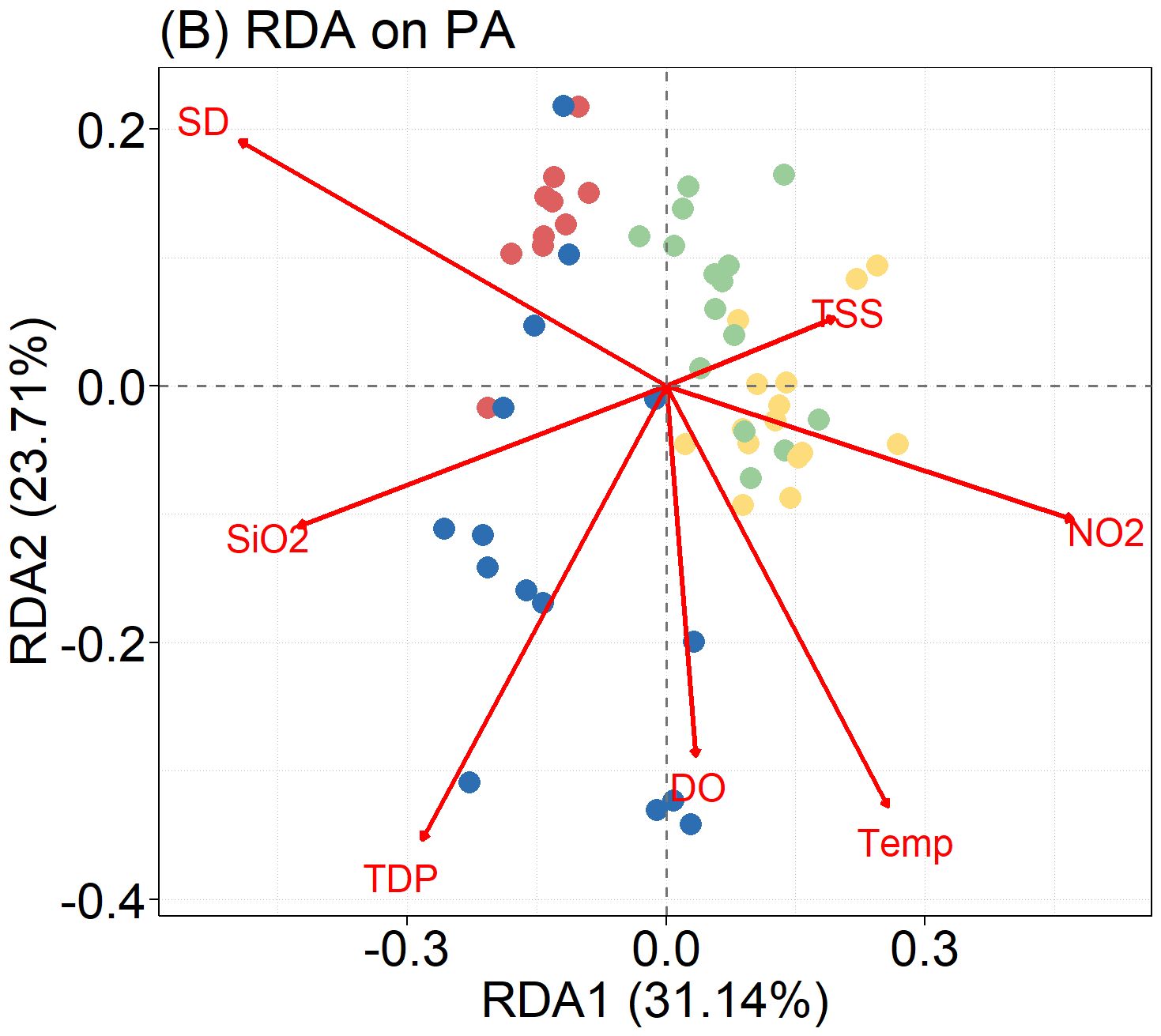
**

**
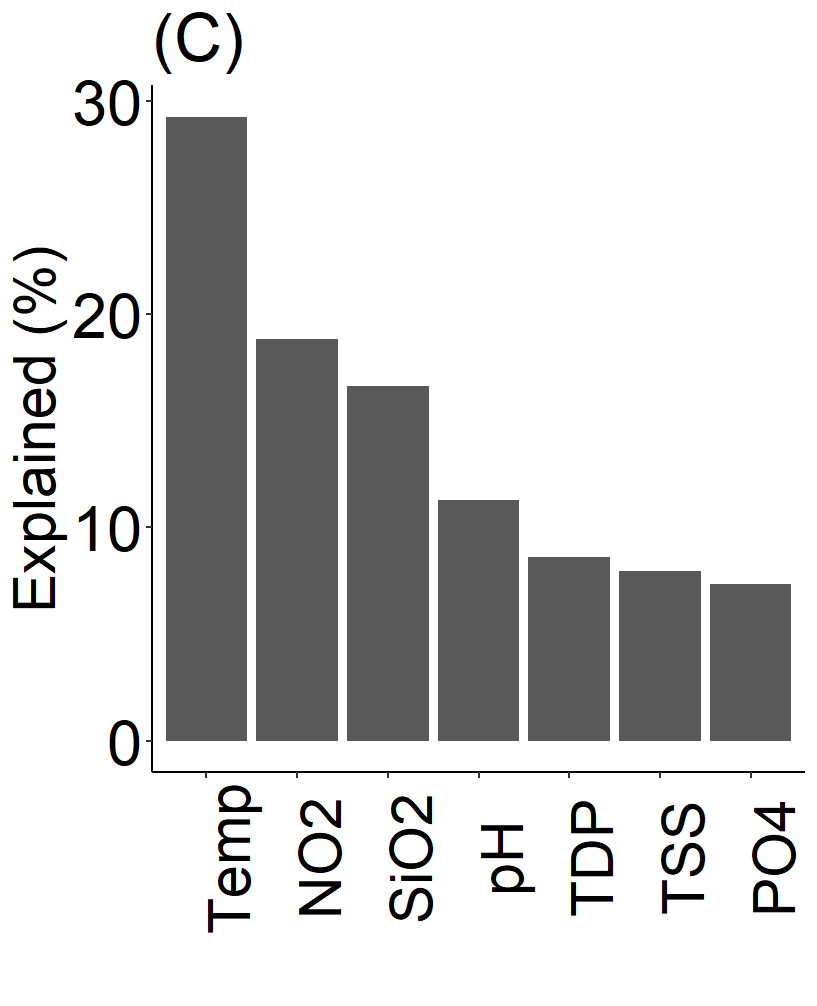

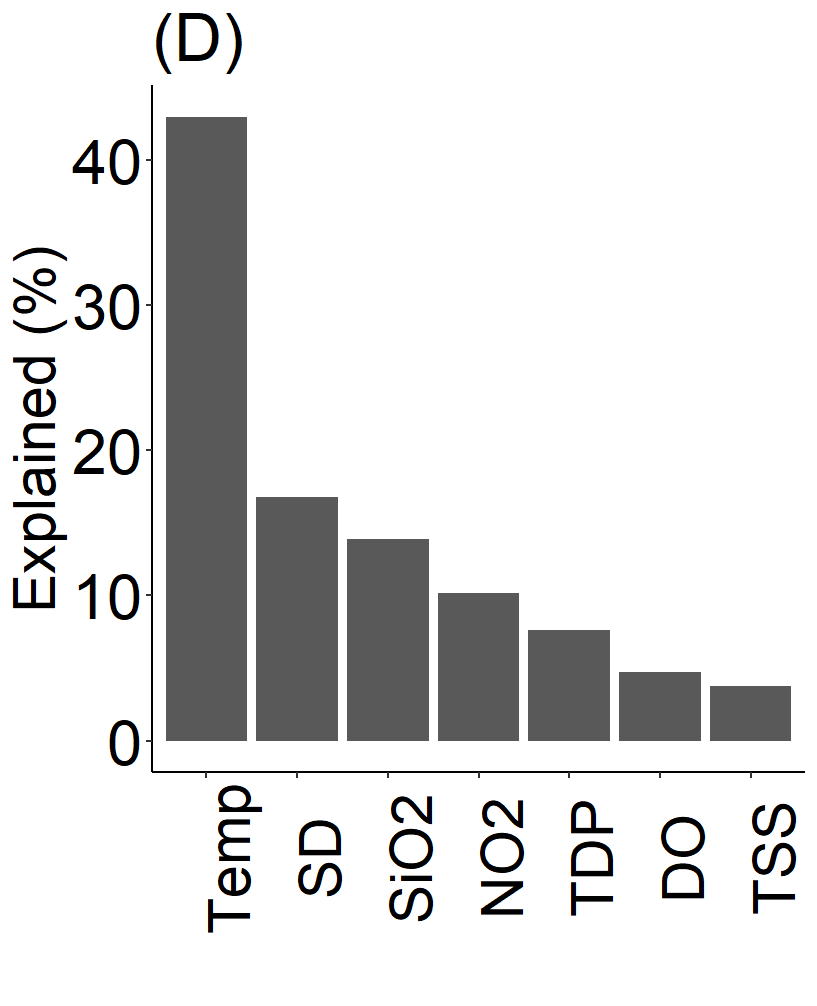
**

Fig. 8 RDA plot (a) BC_FL_ and (b) BC_PA_ and the percentage of variation explained by individual environmental variables for (c) BC_FL_ and (d) BC_PA_
