# Supplementary material for: Seasonal dynamics of free-living (FL) and particle-attached (PA) bacterial communities in a plateau reservoir
Source: Front Microbiol. 2024 Jul 19;15:1428701. doi: 10.3389/fmicb.2024.1428701 (PMC11295932; doi:10.3389/fmicb.2024.1428701)
Supplement: Supplementary file 2 [file Data_Sheet_2.DOCX]

**Supplementary materials 2. R scripts**

## ANOVA on diversity

diversity<-read.csv('diversity.csv')

names(diversity)

data1<-diversity[which(diversity$Group=='FL'),]

data1<-diversity[which(diversity$Group=='PA'),]

names(data1)

fit1<-aov(Richness~Season,data1)

summary(fit1)

fit2<-aov(Simpson~Season,data1)

summary(fit2)

fit3<-aov(Shannon~Season,data1)

summary(fit3)

fit4<-aov(Pielou~Season,data1)

summary(fit4)

fit5<-aov(Chao1~Season,data1)

summary(fit5)

fit6<-aov(ACE~Season,data1)

summary(fit6)

fit7<-aov(PD~Season,data1)

summary(fit7)

## PerMANOVA and PERMDISP

data1<-read.csv('spring.csv',row.names=1)

names(data1)

com_ALL<-data1[,19:ncol(data1)]

fit<-adonis2(com_ALL~Group,data1,perm=999,'bray')

fit

## PERMDISP

dis<-vegdist(com_ALL)

disp<-betadisper(dis,data1$Group)

anova(disp)

plot(disp)

boxplot(disp)

## ANOSIM

fit<-anosim(com_ALL,data1$Group,permutations = 999)

summary(fit)

##RDA

library(tidyverse)

library(vegan)

library(ggpubr)

library(ggrepel)

library(ggplot2)

setwd("D:/r data/PAFL2024/data2")

data0<-read.csv('env_FL.csv',row.names = 1)

names(data0)

otu<-data0[,21:ncol(data0)]

env<-data0[,c(6,7,10,14,15,17,19)]

otu<-decostand(otu,'hellinger')

RDA = rda(otu,env, scale = F)

vif.cca(RDA)

df_rda <- data.frame(RDA$CCA$u[,1:2],rownames(env))

colnames(df_rda)=c("RDA1","RDA2","samples")

df_rda_score <- data.frame(RDA$CCA$v[,1:2])

RDA1 =round(RDA$CCA$eig[1]/sum(RDA$CCA$eig)*100,2)

RDA2 =round(RDA$CCA$eig[2]/sum(RDA$CCA$eig)*100,2)

plotdata<-cbind(df_rda,env)

df_rda_env <- RDA$CCA$biplot[,1:2]

df_rda_env <- as.data.frame(df_rda_env)

Season<-data0$Season

ggplot(plotdata,aes(x=RDA1,y=RDA2,color=Season))+

geom_point(size=6,aes())+

geom_segment(data=df_rda_env,aes(x=0,y=0,xend=df_rda_env[,1]*0.6,yend=df_rda_env[,2]*0.6), arrow = arrow(length = unit(0.01, 'npc')),color="red",size=1.4)+

geom_text(data=df_rda_env,aes(x=df_rda_env[,1]*0.65,y=df_rda_env[,2]*0.65,label=rownames(df_rda_env)),size=8,color="red")+

labs(x=paste0("RDA1 (",RDA1,"%)"),y=paste0("RDA2 (",RDA2,"%)"),title='(A) RDA on FL')+

scale_color_manual(values=c("#2D6DB1","#9BCD9B","#FDDC7B","#DD5F60"))+

geom_hline(aes(yintercept=0), colour="gray45",size=0.8, linetype="dashed")+

geom_vline(aes(xintercept=0), colour="gray45",size=0.8, linetype="dashed")

envfit <- envfit(RDA,env,permutations = 999)

envfit

library(rdacca.hp)

mite.rda.hp <- rdacca.hp(otu, env, method = 'RDA', type = 'adjR2', scale = FALSE)

mite.rda.hp

plot(mite.rda.hp,cex.axis=5,cex.lab=5)

## assembly

library(NST)

library(picante)

library(ape)

library(iCAMP)

library(NST)

otu_FL <- read.csv('otu_FL.csv', row.names = 1)

otu1 <- t(otu_FL)

group <- read.csv('group_FL.csv', row.names = 1)

tree <- read.tree(file='otu.tre')

tree <- prune.sample(otu1, tree)

tree

pd <- cophenetic(tree)

set.seed(123)

qpen.out <- qpen(comm = otu1, pd = pd, sig.bNTI = 2, sig.rc = 0.95, rand.time = 1000, nworker = 8)

write.csv(qpen.out, 'FL_assembly.csv', row.names = FALSE)

otu_AT <- read.csv('otu_PA.csv', row.names = 1)

otu1 <- t(otu_AT)

group <- read.csv('group_PA.csv', row.names = 1)

tree <- read.tree(file='otu.tre')

tree <- prune.sample(otu1, tree)

tree

pd <- cophenetic(tree)

set.seed(123)

qpen.out <- qpen(comm = otu1, pd = pd, sig.bNTI = 2, sig.rc = 0.95, rand.time = 1000, nworker = 8)

head(qpen.out$result)

write.csv(qpen.out, 'PA_assembly.csv', row.names = FALSE)
