# Supplementary material for: Seasonal dynamics of free-living (FL) and particle-attached (PA) bacterial communities in a plateau reservoir
Source: Front Microbiol. 2024 Jul 19;15:1428701. doi: 10.3389/fmicb.2024.1428701 (PMC11295932; doi:10.3389/fmicb.2024.1428701)
Supplement: Supplementary file 3 [file Table_1.docx]

Supplementary materials


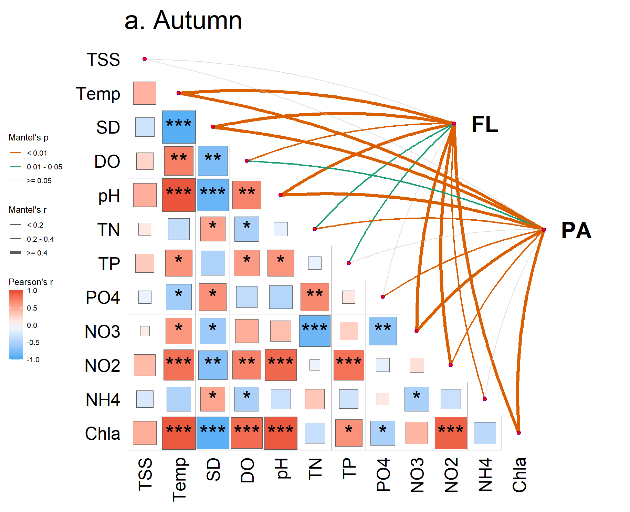

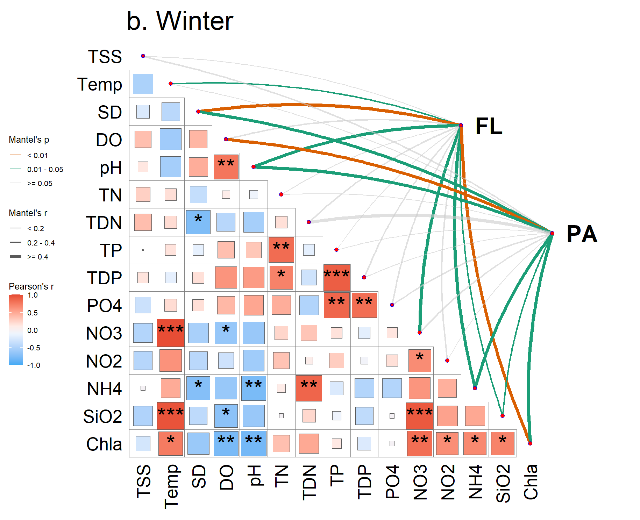

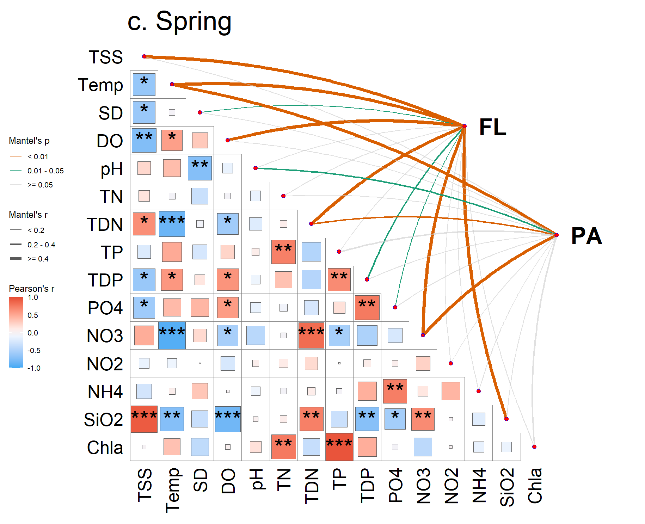

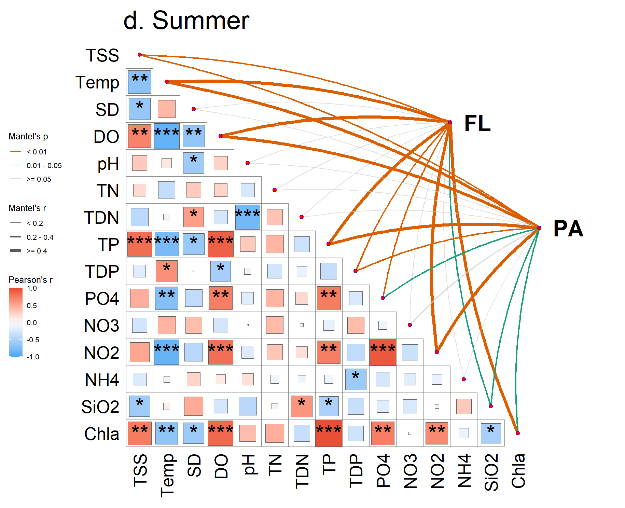


Fig. S1 Mantel tests between FL, PA bacterial communities with environmental factors in Autumn (a), Winter (b), Spring (c), and Summer (d);

Table S1. Topological characteristics of ASV in FL and PA network

| **Lifestyle** | **Node type** | **ASV** | **Phylum** |
| --- | --- | --- | --- |
| FL | Module hubs | ASV_70 | *Cyanobacteriota* |
|  | Connectors | ASV_109 | *Pseudomonadota* |
|  |  | ASV_11 | *Actinomycetota* |
|  |  | ASV_145 | *Actinomycetota* |
|  |  | ASV_155 | *Pseudomonadota* |
|  |  | ASV_159 | *Chloroflexi* |
|  |  | ASV_171 | *Actinomycetota* |
|  |  | ASV_188 | *Actinomycetota* |
|  |  | ASV_2 | *Actinomycetota* |
|  |  | ASV_21 | *Verrucomicrobia* |
|  |  | ASV_264 | *Actinomycetota* |
|  |  | ASV_287 | *Actinomycetota* |
|  |  | ASV_316 | *Actinomycetota* |
|  |  | ASV_339 | *Actinomycetota* |
|  |  | ASV_36 | *Actinomycetota* |
|  |  | ASV_4 | *Pseudomonadota* |
|  |  | ASV_49 | *Bacteroidota* |
|  |  | ASV_51 | *Actinomycetota* |
|  |  | ASV_745 | *Actinomycetota* |
|  |  | ASV_77 | *Actinomycetota* |
|  |  | ASV_89 | *Actinomycetota* |
|  |  | ASV_99 | *Pseudomonadota* |
| PA | Connectors | ASV_1 | *Planctomycetota* |
|  |  | ASV_104 | *Cyanobacteriota* |
|  |  | ASV_11 | *Actinomycetota* |
|  |  | ASV_140 | *Cyanobacteriota* |
|  |  | ASV_203 | *Bacillota* |
|  |  | ASV_21 | *Verrucomicrobia* |
|  |  | ASV_269 | *Cyanobacteriota* |
|  |  | ASV_3 | *Pseudomonadota* |
|  |  | ASV_351 | *Cyanobacteriota* |
|  |  | ASV_36 | *Actinomycetota* |
|  |  | ASV_40 | *Pseudomonadota* |
|  |  | ASV_42 | *Planctomycetota* |
|  |  | ASV_43 | *Cyanobacteriota* |
|  |  | ASV_5 | *Actinomycetota* |
|  |  | ASV_53 | *Planctomycetota* |
|  |  | ASV_7 | *Actinomycetota* |
|  |  | ASV_73 | *Pseudomonadota* |
|  |  | ASV_85 | *Pseudomonadota* |
|  |  | ASV_91 | *Pseudomonadota* |
|  |  | ASV_96 | *Pseudomonadota* |
