# Supplementary material for: Seasonal dynamics of free-living (FL) and particle-attached (PA) bacterial communities in a plateau reservoir
Source: Front Microbiol. 2024 Jul 19;15:1428701. doi: 10.3389/fmicb.2024.1428701 (PMC11295932; doi:10.3389/fmicb.2024.1428701)
Supplement: Supplementary file 4 [file Table_2.DOCX]

**Tables**

Table 1. PerMANOVA, ANOSIM and PERMDISP tests between BC_FL_ and BC_PA_ within each season.

| Season | Adonis | ANOSIM | PERMDISP |
| --- | --- | --- | --- |
| Spring | *0.001* | *0.001* | 0.09 |
| Summer | *0.001* | *0.001* | 0.11 |
| Autumn | *0.001* | *0.001* | *<0.001* |
| Winter | *0.001* | *0.001* | *0.003* |

Table 2. Topological attributes of co-occurrence network of BC_FL_ and BC_PA_ bacteria in the Wujiangdu reservoir.

|  | Node | Edge | | Modularity | Density | AD | avgPL | avgCC |
| --- | --- | --- | --- | --- | --- | --- | --- | --- |
| FL | 90 | 1396/432 |  | 0.15 | 0.35 | 31.02 | 1.67 | 0.46 |
| PA | 57 | 437/199 |  | 0.20 | 0.27 | 15.33 | 1.78 | 0.41 |

(AD: average degree; avgPL: average path; avgCC: average clustering coefficient)
